# Supplementary material for: Avian-inspired embodied perception in biohybrid flapping-wing robotics
Source: Nat Commun. 2024 Oct 22;15:9099. doi: 10.1038/s41467-024-53517-6 (PMC11496644; doi:10.1038/s41467-024-53517-6)
Supplement: Supplementary file 1 — Supplementary Information [file 41467_2024_53517_MOESM1_ESM.pdf]

Avian-Inspired Embodied Perception in Biohybrid Flapping-Wing Robotics

Qian Li[1], Ting Tan [2]*, Benlong Wang[1], Zhimiao Yan [1]*

1. State Key Laboratory of Ocean Engineering, Department of Engineering Mechanics, School of Ocean and Civil Engineering, Shanghai Jiao Tong University, Shanghai 200240, China.

2. State Key Laboratory of Mechanical System and Vibration, School of Mechanical Engineering, Shanghai Jiao Tong University, Shanghai 200240, China.

* Corresponding Author.

E-mail address: zhimiaoy@sjtu.edu.cn (Zhimiao Yan), tingtan@sjtu.edu.cn (Ting Tan)

This PDF file includes:

Supplementary Fig. 1: Schematic illustration of the working principle of piezoelectric thin films

Supplementary Fig. 2: Microscopic characterization of feathers by SEM

Supplementary Fig. 3: Characterization of PVDF material properties

Supplementary Fig. 4: Microscopic characterization of feather-PVDF adhesion

Supplementary Fig. 5: Localized waveform and spectral analysis of voltage during dynamic fatigue testing

Supplementary Fig. 6: Flapping mechanism structure

Supplementary Fig. 7: Flapping mechanism kinematic parameters

Supplementary Fig. 8: Experimental measurement devices

Supplementary Fig. 9: Wind tunnel devices

Supplementary Fig. 10: Output performance of the feather-PVDF bio-hybrid sensor

Supplementary Fig. 11: Voltage signals and spectrograms of a single feather at various flapping frequencies

Supplementary Fig. 12: Voltage signals and spectrograms of a single feather at various wind speeds (without flapping)

Supplementary Fig. 13: Voltage signals and spectrograms of multi-feather structures at various wind speeds (without flapping)

Supplementary Fig. 14: Voltage signals and spectrograms of multi-feather structures at various flapping frequencies

Supplementary Fig. 15: Voltage signals and spectrograms of multi-feather structures flapping at 3.5Hz under various wind speeds

Supplementary Fig. 16: Voltage signals and spectrograms of multi-feather structures flapping at 2.5Hz with $3 m\cdot s^{-1}$ wind speeds under various pitch angles

Supplementary Fig. 17: Flapping wing kinematic model

Supplementary Fig. 18: Simulation of load magnitude on a single wing under various operating conditions

Supplementary Fig. 19: Forced vibrational response of linear systems

Supplementary Fig. 20: Forced vibrational response of systems with cubic nonlinearities

Supplementary Fig. 21: Forced vibrational response of systems with quadratic nonlinearities

Supplementary Fig. 22: Response of systems with second and third-order nonlinearities to forced vibrations

Supplementary Fig. 23: Resonance response of systems with second and third-order nonlinearities

Supplementary Fig. 24: Root mean square error (RMSE) in flapping frequency identification across diverse sample input dimensions

Supplementary Fig. 25: Fitness iteration graph of the grey golf optimizer and iteration graph of the optimization network training loss function

Supplementary Fig. 26: Histogram of training errors for convolutional neural network

Supplementary Fig. 27: Raw signals generated by variable-frequency flapping

Supplementary Fig. 28: Flapping frequency recognition results at different sliding window step sizes

Supplementary Fig. 29: Display and parameter description of the feathered flapping wing robot

Supplementary Fig. 30: Generalization error in variable-frequency flight of the feathered flapping wing robot after transfer learning

Supplementary Fig. 31: Generalization results and errors in variable-frequency flight of the feathered flapping wing robot recognized by the original network without transfer learning

Supplementary Fig. 32: The principal component distribution, obtained through principal component analysis (PCA), exhibits a cumulative explained variance contribution rate exceeding 95%

Supplementary Fig. 33: Operational principles of the flapping-wing robot

Supplementary Fig. 34: Schematic diagram of the component connections in the drive module

Supplementary Fig. 35: Integrated signal acquisition and wireless transmission module

Supplementary Fig. 36: The motion capture system

Supplementary Fig. 37: Comparison of flapping frequency monitored by the laser displacement sensor with the signal's dominant frequency

Supplementary Table 1: The hyperparameters of the neural network for flapping frequency recognition

Supplementary Table 2: The hyperparameters of the neural network for wind speed recognition

Supplementary Table 3: The hyperparameters of the neural network for pitch angle recognition

Supplementary Table 4: Comparative analysis of parameter identification error in our work versus other fixed-wing studies

Supplementary Table 5: The variables for feature extraction

Supplementary Table 6: Mass decomposition chart for feathered flapping-wing robots

Supplementary References (1-13)

Supplementary Movie 1: Feather-PVDF biohybrid perceptual structure introduction

Supplementary Movie 2: Peeling experiment

Supplementary Movie 3: Fatigue experiment

Supplementary Movie 4: Bending experiment

Supplementary Movie 5: Motion and environmental perception experiments

Supplementary Movie 6: The data preprocessing, training methodology and presentation of results

Supplementary Movie 7: The real-time identification of flapping frequency in feathered flapping-wing robot

Supplementary Movie 8: The real-time identification of wind speed in feathered flapping-wing robot

Supplementary Movie 9: The real-time identification of pitch angle in feathered flapping-wing robot

Supplementary Movie 10: The real-time identification of wing shape in feathered flapping-wing robot

Supplementary Movie 11: Experiments of untethered indoor and outdoor flight

1. The working principle of piezoelectric materials

When a piezoelectric material undergoes deformation due to external forces, polarization occurs internally. This excites movement of positive and negative charge centers within the material, resulting in opposite polarity charges of equal magnitude on its upper and lower surfaces, and generating an electric field within. Upon removal of the external force, the piezoelectric material gradually returns to its original undeformed state, causing the electric field strength to diminish until the charged state disappears. Reversal of external force causes a corresponding reversal in the movement of positive and negative charges. Piezoelectric sensors utilize the direct piezoelectric effect principle, where electrodes connected to the upper and lower surfaces of the material form a circuit. When the sensor undergoes deformation due to external forces, voltage changes can be detected to reflect the applied load (Supplementary Fig. 1).

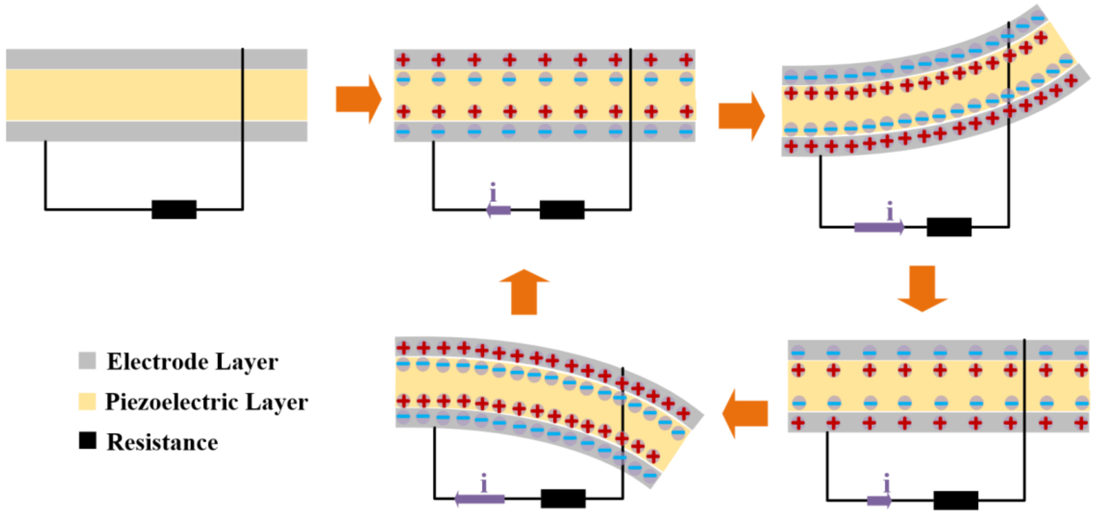

Supplementary Fig. 1: Schematic illustration of the working principle of piezoelectric thin films.

2.Microscopic characterization of materials

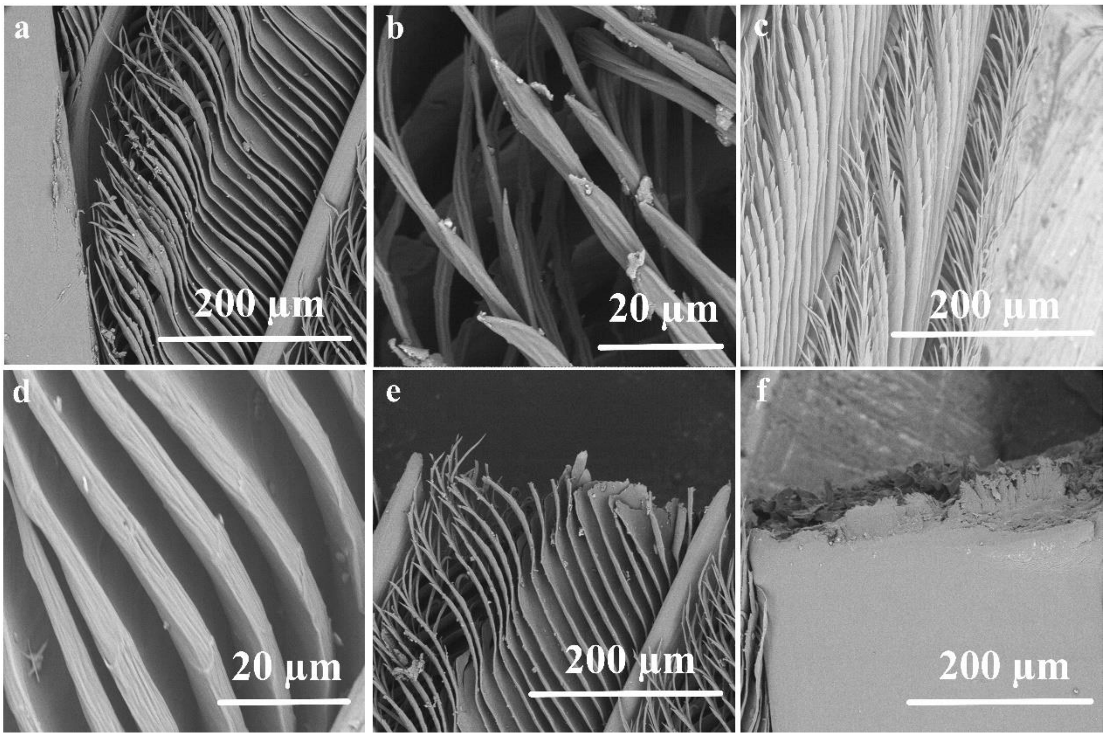

Supplementary Fig. 2: Scanning electron microscopy (SEM) micro characterization of feathers. a Microscopic characterization of feather barbs between adjacent barbules. **b** Microscopic characterization of barbules with hooks. **c** Microscopic characterization of the feather edge. **d** Microscopic characterization of barbs and barbules. **e** Microscopic characterization of the fracture surface of barbs and barbules. **f** Microscopic characterization of the fracture surface of the rachis.

Utilizing a low-vacuum, ultra-high-resolution field emission scanning electron microscope (NOVA Nano SEM 230, USA), we observed the microstructure of the feather surface. The feather is composed of two parallel rachises, and on either side of the rachis, there are parallel-arranged barbs with different structures. The barbs on the side facing the feather tip have numerous hooks extending from the midsection to the ventral side, referred to as hooked barbules. The cascaded slide-lock system[1], consisting of hooks, a groove, and cascading barbules at the groove's end, ensures strong adhesion between barbs and protects the barbs from damage during separation.

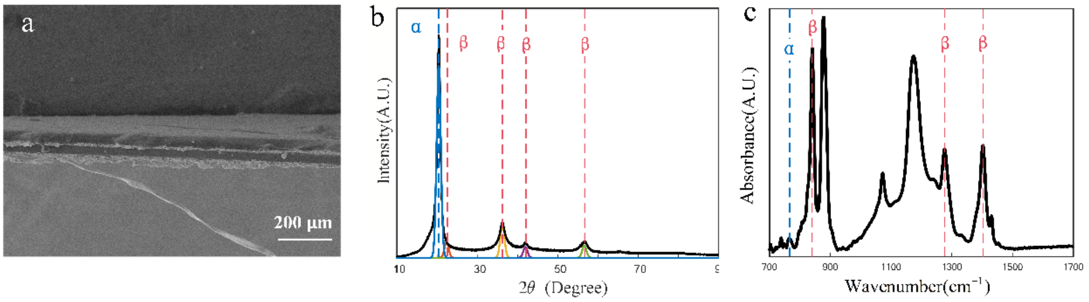

Supplementary Fig. 3: Characterization of PVDF material properties. a Scanning electron microscope (SEM) morphology of the PVDF film. b X-ray diffraction (XRD) analysis of the PVDF sample. **c** Fourier-transform infrared spectroscopy (FTIR) analysis of the PVDF sample.

The PET used in this study was purchased from Shenzhen Guangzhongtai Industrial Co., Ltd., with a thickness of 0.1 mm. The carbon fiber sheets were obtained from Yangzhou Falbo Carbon Fiber Products Co., Ltd., with a thickness of 0.1 mm. The PVDF film was procured from Shenzhen Aimin Intelligent Technology Co., Ltd., with the model FLDT1-028K. The sensing part of the PVDF film is a rectangular piezoelectric film component measuring 17.65 mm × 31.18 mm, featuring silver ink-printed electrodes. One end of the narrow, flat lead is connected to the sensing film, while the other end is connected to a connector for pin connection. The total length of the component is 145.51 mm, and the total thickness is 125 μm, with a piezoelectric layer thickness of 28 μm and a capacitance of 1.37 nF. Feathers were purchased from Shenzhen Xiaoqiao Shidai Information Consulting Co., Ltd., with an average diameter of about 4 mm and an average length of approximately 12-14 cm. As an electroactive polymer, PVDF exhibits strong piezoelectric properties. We observed the scanning electron microscope (SEM) morphology of the PVDF film, showing evident layering (Supplementary Fig. 3a). Among all stable crystal variants (α, β, γ, and δ phases), the polar β-phase demonstrates superior piezoelectric performance[2]. X-ray diffraction (XRD) and Fourier-transform infrared spectroscopy (FTIR) analyses were conducted on the PVDF samples. Observing the XRD spectrum (Supplementary Fig. 3b), the

diffraction peak at 20.43° corresponds to the non-polar α-phase (110) crystal plane[2]. Peaks at 22.40°, 36.29°, 41.83°, and 56.43° belong to the polar β-phase, with the 22.40° peak corresponding to the (110) and (200) planes of the β-phase, and the peaks at 36.29°, 41.83°, and 56.43° corresponding to the (001), (201), and (221) planes[3] of the β-phase, respectively. From the FTIR results (Supplementary Fig. 3c), the absorption peak at 763 cm^{-1} belongs to the non-polar α-phase[3]. The peaks at 840 cm^{-1}, 1278 cm^{-1}, and 1400 cm^{-1} in the FTIR vibration spectrum are attributed to the polar β-phase. The relative fraction of the β-phase can be calculated using the following formula[4]:

$$F_{\beta}=\frac{A_{840}}{\left(\frac{K_{840}}{K_{763}}\right)A_{763}+A_{840}}\times 100\% \qquad (1)$$

Where A_{763} and A_{840} are the absorbances at 763 cm^{-1} and 840 cm^{-1}, respectively, and K_{763} and K_{840} are the respective absorption coefficients at these wavenumbers:

$$K_{763}=6.1\times10^{4}\ cm^{2}\ mol^{-1}$$

$$K_{840}=7.7\times10^{4}\ cm^{2}\ mol^{-1}$$

Therefore, the relative fraction of the β-phase is 80.6%, demonstrating commendable piezoelectric performance.

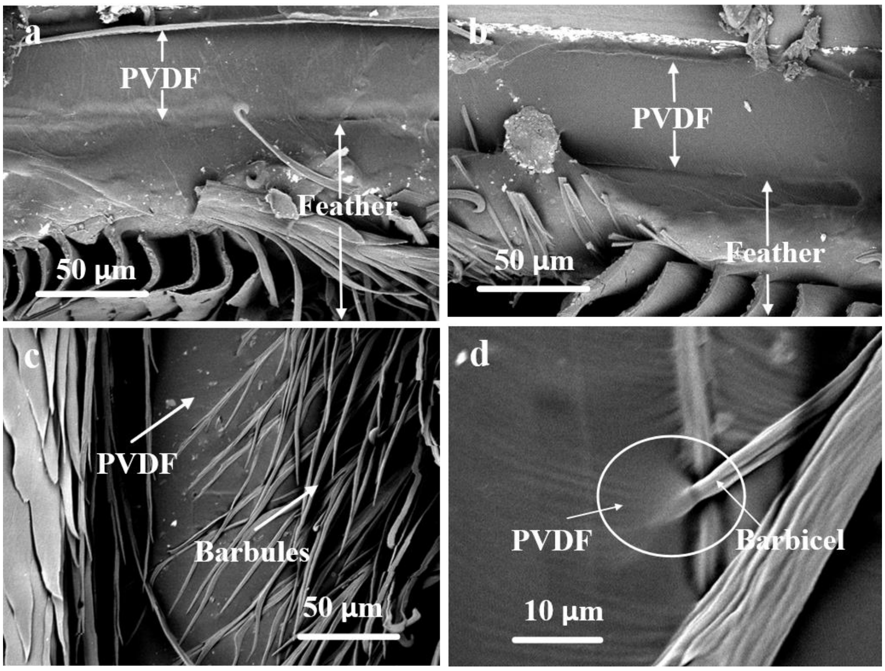

Supplementary Fig. 4: Microscopic characterization of feather-PVDF adhesion. a and b Microscopic characterization of the cross-sectional adhesion. **c** Microscopic characterization of the adhesion surface. **d** Local magnification of the adhesion surface microscopic features.

3.Signals analysis during dynamic fatigue testing

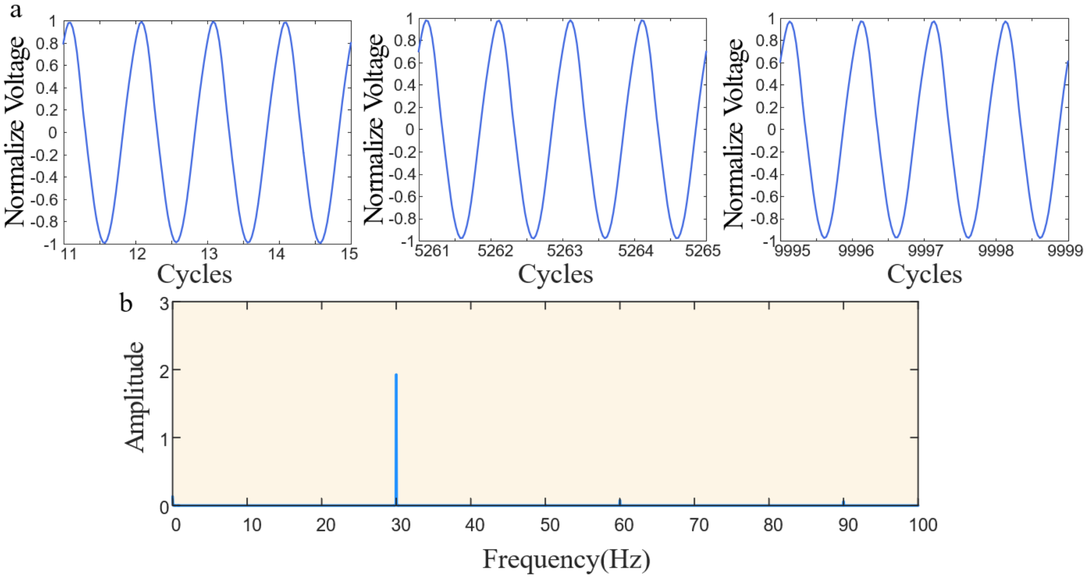

Supplementary Fig. 5: Localized waveform and spectral analysis of voltage during dynamic fatigue testing. a Localized voltage signals in the initial (11-15), intermediate (5261-5265), and terminal (9995-9999) segments. **b** Spectral analysis of normalized voltage signals under 10,000 cycles of loading. Source data are provided as a Source Data file.

4.Piezoelectric cantilevered beam electromechanical coupling model

The feather-PVDF structure is equivalent to a piezoelectric cantilevered beam model[5,6], establishing a distributed parameter model based on the Euler-Bernoulli beam theory. Initially, an analysis is conducted on the undamped free vibration of the piezoelectric cantilevered beam under no external load excitation, and the motion equation is formulated as follows:

$$EI\frac{\partial^4\mathbf{v}(x,t)}{\partial x^4}+m\frac{\partial^2\mathbf{v}(x,t)}{\partial t^2}=0 \tag{1}$$

Here, $\mathbf{v}(x,t)$ represents the lateral deformation of the cantilever beam, EI is the bending stiffness, and m is the distributed mass.

Let:

$$\mathbf{v}(x,t)=\phi(x)\mathrm{e}^{i\omega t} \tag{2}$$

Here, $\phi(x)$ represents the modal shape of the piezoelectric beam, and ω is the vibration frequency. Substituting these values into the equation, the fundamental solution of the equation can be obtained:

$$\phi(x)=A\sin\beta x+B\cos\beta x+C\sinh\beta x+D\cosh\beta x \tag{3}$$

Here, $\beta=\sqrt[4]{\omega^2 m\,/\,EI}$, By substituting the fundamental solution into the boundary conditions and continuity conditions, solving the system of equations yields the vibration frequencies and modal shapes of the piezoelectric beam.

Considering electromechanical coupling, the introduction of structural damping, piezoelectric effect, and external load terms. The voltage generated by the piezoelectric material's vibration will impose an additional moment on the cantilever beam:

$$\mathbf{M}(x,t)=EI_1\frac{\partial^2\mathbf{v}_1(x,t)}{\partial x^2}+\theta V(t)[H(x)-H(x-L_1)] \tag{4}$$

Where θ represents the coupling term, H(x) is the Haversine function. $\mathbf{v}_1(x,t)$ and EI_1denote the lateral deformation and bending stiffness of the piezoelectric segment cantilever beam, $V(t)$ is the voltage across the load resistor, and L_1 is the length of the piezoelectric segment cantilever beam.

Building on the piezoelectric constitutive equation, we establish the circuit equation incorporating the electromechanical coupling terms. For the case of a single piezoelectric element in a cantilever beam vibration, the relationship between the electrical displacement vector and the strain and voltage is given by:

$$D_3(x,t) = -d_{31}E_p \frac{h_p+h_s}{2}\frac{\partial^2 \mathbf{v}(x,t)}{\partial x^2} - \varepsilon_{33}^S \frac{V(t)}{h_p} \tag{5}$$

Here, ε_{33}^S is the dielectric constant of the piezoelectric material under constant strain conditions, E_p is the elastic modulus of piezoelectric materials, h_p and $h_s$represent the thickness of the piezoelectric material and the beam, respectively, and d_{31} is the piezoelectric constant.

The output current is:

$$I_t = \frac{\mathrm{d}}{\mathrm{d}t}\left[\int_0^{l_1} b_P D_3(x,t)\,\mathrm{d}x\right] = \theta \int_0^{L_1} \frac{\partial^3 \mathbf{v}(x,t)}{\partial x^2 \partial t}\mathrm{d}x - C_p \frac{\mathrm{d}V(t)}{\mathrm{d}t} \tag{6}$$

Here, C_p represents the capacitance of the piezoelectric material. b_P is the width of the piezoelectric material.

When the load resistance is R, the coupled circuit equation is:

$$\frac{V(t)}{R} + C_P \frac{\mathrm{d}V(t)}{\mathrm{d}t} - \theta \int_0^{L_1} \frac{\partial^3 \mathbf{v}(x,t)}{\partial x^2 \partial t}\mathrm{d}x = 0\,, 0 < x < L_1 \tag{7}$$

Utilizing the Galerkin method, the displacement of the cantilever beam is discretized and expressed in the form of the product of modal equations and modal coordinates, i.e.:

$$\mathbf{v}(x,t) = \Sigma \phi_r(x) q_r \tag{8}$$

Here, q_r represents the modal coordinates of the natural frequency of the composite beam.

Solely considering the first-order mode, we can derive the coupled electromechanical control equations:

$$\ddot{q}(t) + 2\xi\omega_n \dot{q}(t) + \omega_n^2 q(t) + \theta_p V(t) = F_t \tag{9}$$

$$\frac{V(t)}{R} + C_P\dot{V}(t) - \theta_p\dot{q}(t) = 0 \tag{10}$$

Where ξ is the damping ratio, ω_n is the natural frequency, and θ_p is the electromechanical coupling factor, expressed as:

$$\theta_p = \theta[\phi_1'(L_1) - \phi_1'(0)]$$

The solution to the control equation can take the form of a superposition of multiple harmonic components. In the presence of forced vibration, the frequency components of the solution are typically the excitation frequency and its harmonics. We assume that the solution to the control equation is:

$$q(t) = \frac{1}{2}a_1e^{i\Omega t} + \mathrm{cc} \tag{11}$$

$$V(t) = \frac{1}{2}a_2e^{i\Omega t} + \mathrm{cc} \tag{12}$$

Here, a_1 and a_2 represent the wing displacement amplitude and output voltage, respectively, while Ω denotes the frequency of modal coordinates and voltage, and cc represents the complex conjugate. Since displacement and output voltage are also superpositions of multiple waves, the amplitude and frequency here can be considered in a general sense. The voltage amplitude can be expressed as a function of modal coordinate amplitudes,

$$a_2 = \frac{\theta_p\Omega R}{C_p\Omega R - i}a_1 \tag{13}$$

Hence, the output voltage is obtained as follows:

$$V(t) = \frac{1}{2}\frac{\theta_p\Omega R}{C_p\Omega R - i}a_1e^{i\Omega t} + \mathrm{cc} \tag{14}$$

Here, the generalized Ω encompasses frequency components associated with the wing frequency excitation. As the flapping frequency increases, the frequency and amplitude of the output voltage also increase accordingly.

5.Introduction to flapping mechanism and motion parameters

We implemented the flapping motion using the mechanism assembly shown in Supplementary Fig. 5a. This assembly includes a gear set (Supplementary Fig. 6b), an outer shell (yellow part), flapping rods (silver part), and a DC motor (rated voltage: 12V). The drive mechanism is fixed on a 3D-printed support platform. A carbon fiber 3D-printed interface device is used to connect the feathers and the drive mechanism. One end of this device is inserted into the metal cylinder of the drive mechanism, and the other end has six 4mm-diameter holes for feather insertion. Each wing is composed of six feathers stacked sequentially on each side. After assembling the wing structure, tape is used to wrap around the roots of the six feathers on both sides and the metal rod of the drive mechanism to maintain their fixed relative positions.

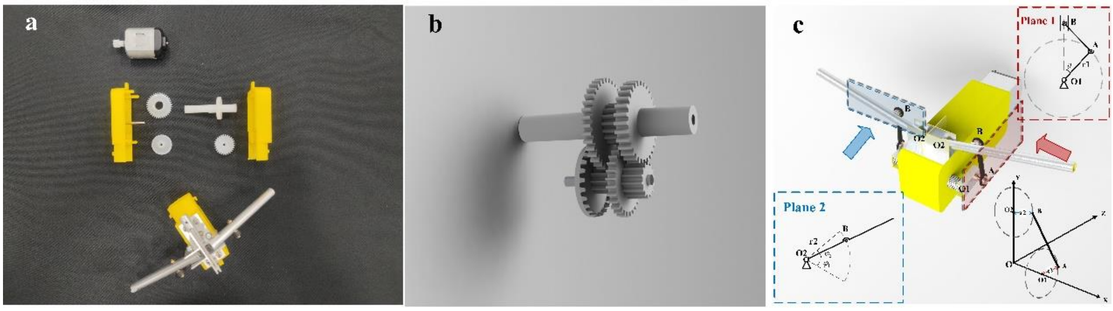

Supplementary Fig. 6: Flapping mechanism structure. a Physical representation of the gear set, motor, and outer shell. **b** Schematic diagram of the gear set structure. **c** Analytical illustration of the flapping mechanism.

As shown in Supplementary Fig. 6c, we analyze the kinematics of the mechanism from both a front view (Plane 1, blue) and a side view (Plane 2, red). In Plane 1, point A undergoes circular motion in the plane with O_1 as the center, forming a circle with the radius AO_1 of 1.5 cm. Point A is connected to point B by a straight rod, AB, with a length of 2.8 cm, and both points A and B serve as spherical pairs. In Plane 2, point B undergoes arc motion in the plane with O_2 as the center, forming an arc with the radius BO_2 of 1.7 cm. The upstroke flapping angle is $\varphi_1 = 40°$, and the downstroke flapping angle is $\varphi_2 = 30°$. Rod AO_1 serves as the driving link.

Establishing a three-dimensional coordinate system as depicted in Supplementary

Fig. 6c, where the coordinates of point A are (x_A, y_A, z_A) and those of point B are (x_B, y_B, z_B). The distances OO_1 and OO_2 are denoted as L_1 and L_2, respectively. The distance from A to O_1 is represented as r_1, from B to O_2 as r_2, and the length of AB as L_{AB}. The rotational angle of rod AO_1 is θ_1, with an angular velocity of $\boldsymbol{\omega_1}$, and the rotational angle of rod BO_2 is θ_2, with an angular velocity of $\boldsymbol{\omega}_2$.

Let:

$$A: \{\, x_A = L_1 \,;\; y_A = r_1 \sin\theta_1 \,;\; z_A = r_1 \cos\theta_1 \,\} \tag{1}$$

$$\mathrm{B}: \{\, x_B = r_2 \sin\theta_2 \;;\; y_B = r_2 \cos\theta_2 + L_2 \,;\; z_B = 0 \,\} \tag{2}$$

Based on geometric relationships, the following expressions can be derived:

$$(x_A - x_B)^2 + (y_A - y_B)^2 + (z_A - z_B)^2 = L_{\mathrm{AB}}^2 \tag{3}$$

Substituting the above coordinates and simplifying, we obtain:

$$\theta_1 = \arcsin\left(\frac{C + a_3 \cos\theta_2 - a_4 \sin\theta_2}{a_1 \cos\theta_2 + a_2}\right) \tag{4}$$

Here, $a_1 = 2r_1r_2$, $a_2 = 2r_1L_2$, $a_3 = 2r_2L_2$, $a_4 = 2L_1r_2$, $C = L_1^2 + L_2^2 + r_1^2 + r_2^2 - L_{\mathrm{AB}}^2$.

Differentiating with respect to time, we obtain:

$$\boldsymbol{\omega}_1 = \frac{Ca_1 \sin\theta_2 - a_2a_3 \sin\theta_2 - a_2a_4 \cos\theta_2 - a_1a_4}{(a_1 \cos\theta_2 + a_2)^2 \cos\theta_1} \boldsymbol{\omega}_2 \tag{5}$$

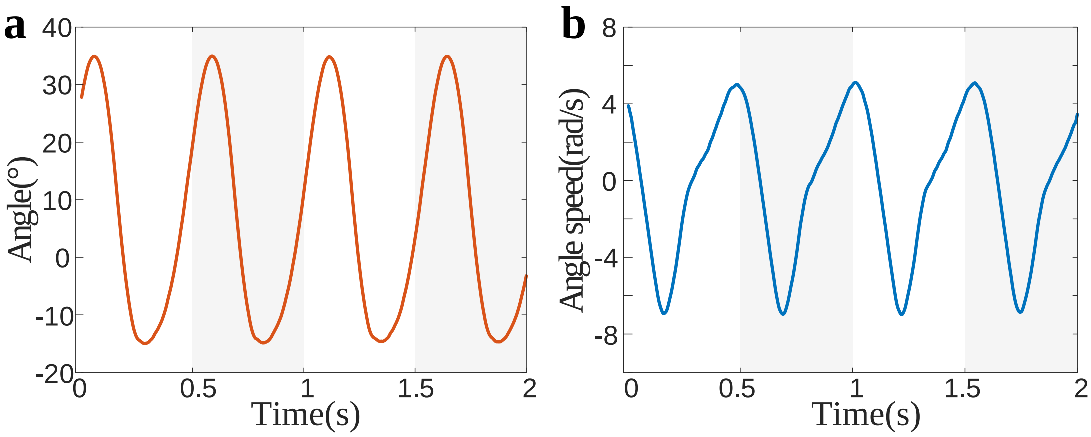

Supplementary Fig. 7: Flapping mechanism kinematic parameters. a Stroke angle curve of the flapping mechanism. **b** Angular velocity curve of the flapping mechanism. Source data are provided as a Source Data file.

6.Motion and environmental perception

The system was powered by a 12V direct current power supply, with speed control facilitated by a digital speed controller. One end of the digital speed controller was connected to the 12V power source, while the other end was linked to the motor pins of the drive mechanism. By activating the power supply and adjusting the digital speed controller knob within the displayed range (0-100), motor speed could be regulated. To monitor the flapping motion, a KEYENCE IL-300 laser displacement sensor (Supplementary Fig. 8a) was employed. The infrared laser emitter was fixed above the drive mechanism, directing the laser onto a retro-reflective tape. The sensor amplifier connected to the infrared laser emitter detected changes in vertical position between the retro-reflective tape and the infrared laser emitter, generating a corresponding voltage output. PVDF piezoelectric sheets were affixed to specific feathers, and the lead wires from the piezoelectric sheets and the output leads from the sensor amplifier were simultaneously connected to a data acquisition system (East China DH5922D) for voltage signal acquisition. Adjustment of the pitch angle was achieved using a tilting platform. The support platform with the drive mechanism was secured to the tilting platform, and the pitch angle of the flapping mechanism could be altered by turning the knob on the tilting platform. The wind tunnel, located at the Engineering Mechanics Experiment Center of Shanghai Jiao Tong University, is an open-circuit type with a total length of 4.71 meters and a width of 1.02 meters. It comprises an expansion section, settling chamber, contraction section, test section, and exhaust duct. The test section (Supplementary Fig. 9) is 0.60 m in length with a square cross-section having a side length of 0.33 m and an inner wall thickness of 15 mm. The top features a removable rectangular cover plate measuring 0.46 m in length and 0.24 m in width. The tunnel features six layers of honeycomb and mesh screens for flow straightening and is powered by a variable-frequency, three-phase asynchronous motor with a rated power of 7.5 kW and a rated voltage of 380V, and the actual wind speed was measured by a JAHAN W410D2 split-type anemometer (Supplementary Fig. 8b).

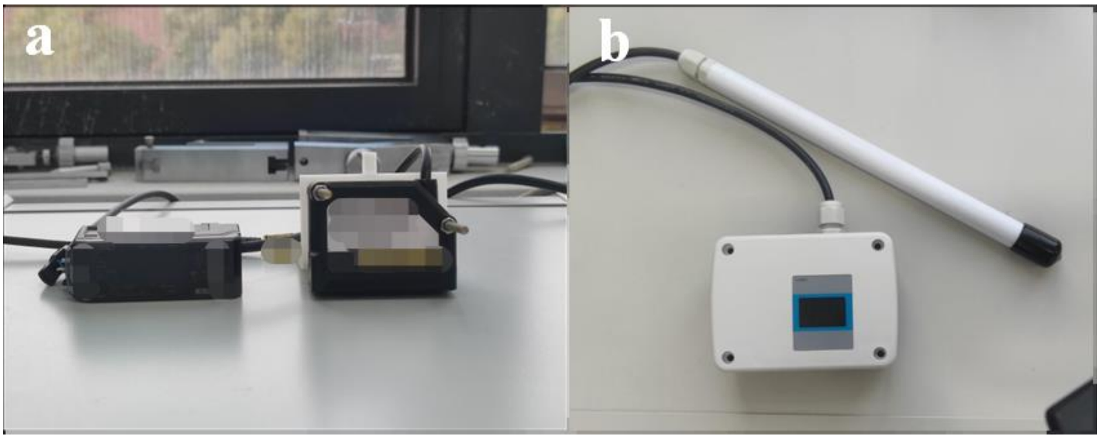

Supplementary Fig. 8: Experimental measurement devices. a Laser displacement sensor. **b** Anemometer.

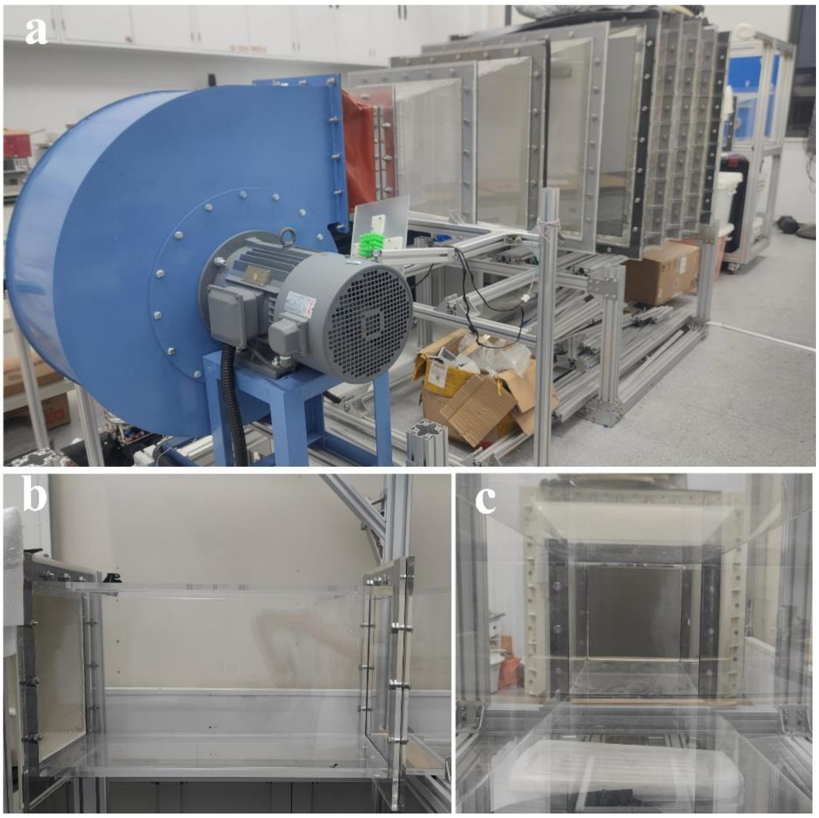

Supplementary Fig. 9: Wind tunnel devices. a Overall structure of the wind tunnel. **b** Wind tunnel test section. **c** Internal structure of the wind tunnel.

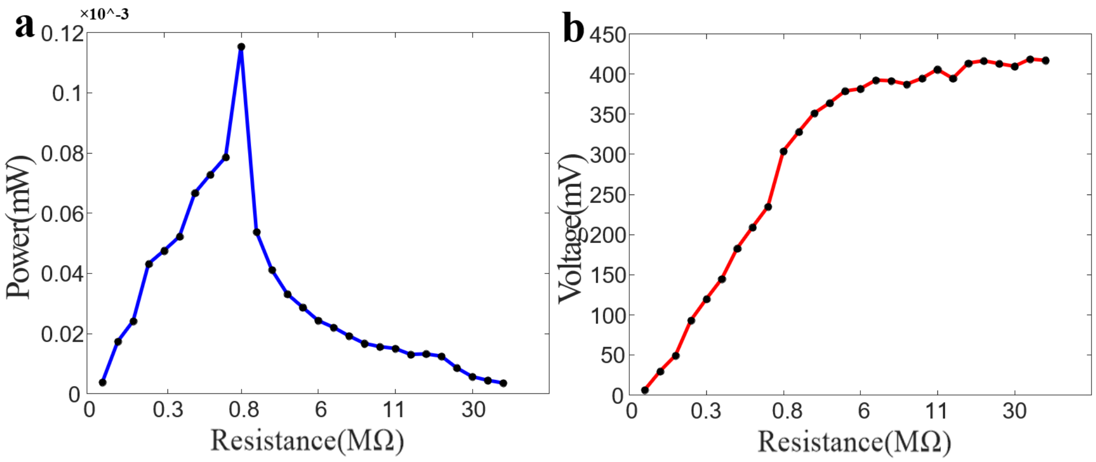

Supplementary Fig. 10: Output performance of the feather-PVDF bio-hybrid sensor. a Output power of the feather-PVDF bio-hybrid sensor under different load resistances. **b** RMS output voltage of the feather-PVDF bio-hybrid sensor under different load resistances. Source data are provided as a Source Data file.

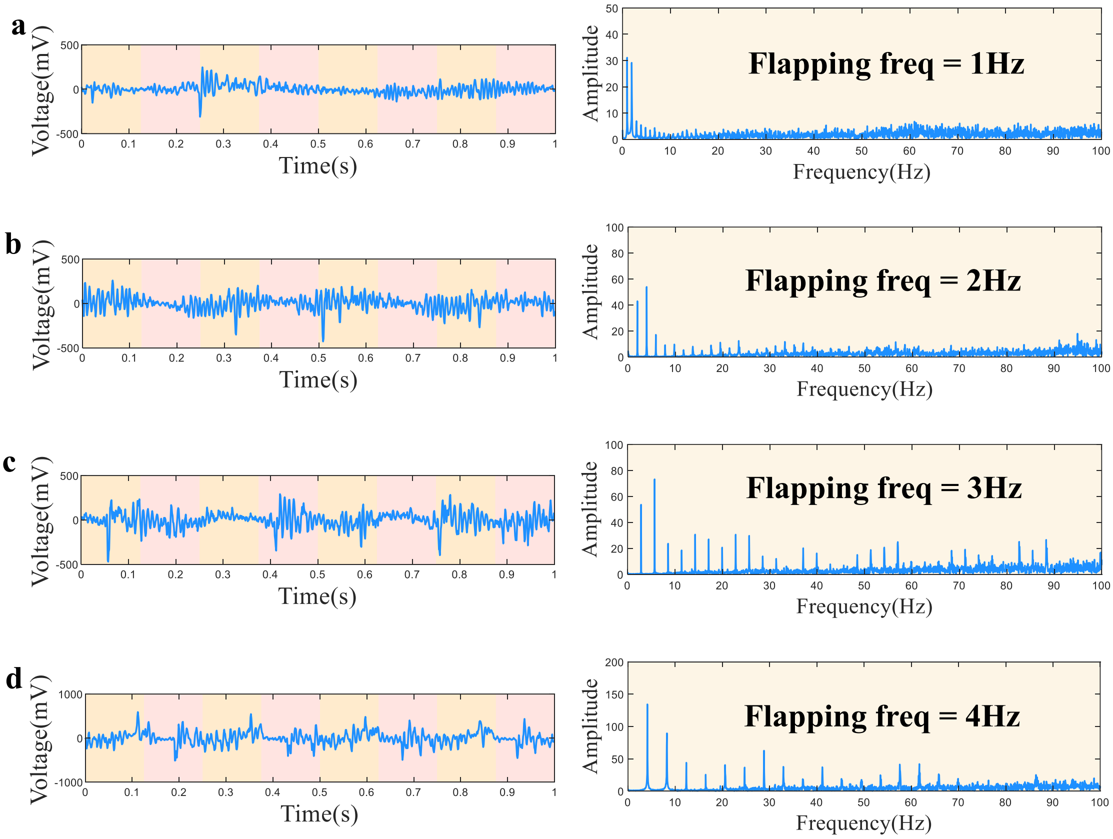

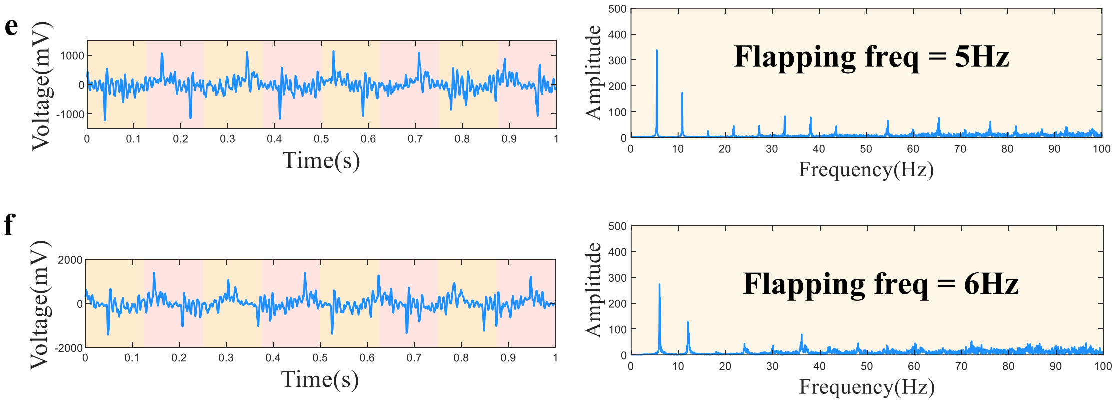

Supplementary Fig. 11: Voltage signals and spectrograms of a single feather at various flapping frequencies.

Utilizing a single feather mounted on the flapping mechanism depicted in Supplementary Fig. 6, flapping signals were collected across frequencies ranging from 1 Hz to 6 Hz with an increment of 1 Hz. Fast Fourier Transform (FFT) was employed for spectral analysis. As the flapping frequency increased, the amplitude of the time-domain curve also increased. The spectral analysis revealed dominant frequencies, including the fundamental frequency and its harmonics. The amplitude of the fundamental frequency exhibited an increasing trend. Additionally, complex, widely distributed noise was observed in the higher frequency range.

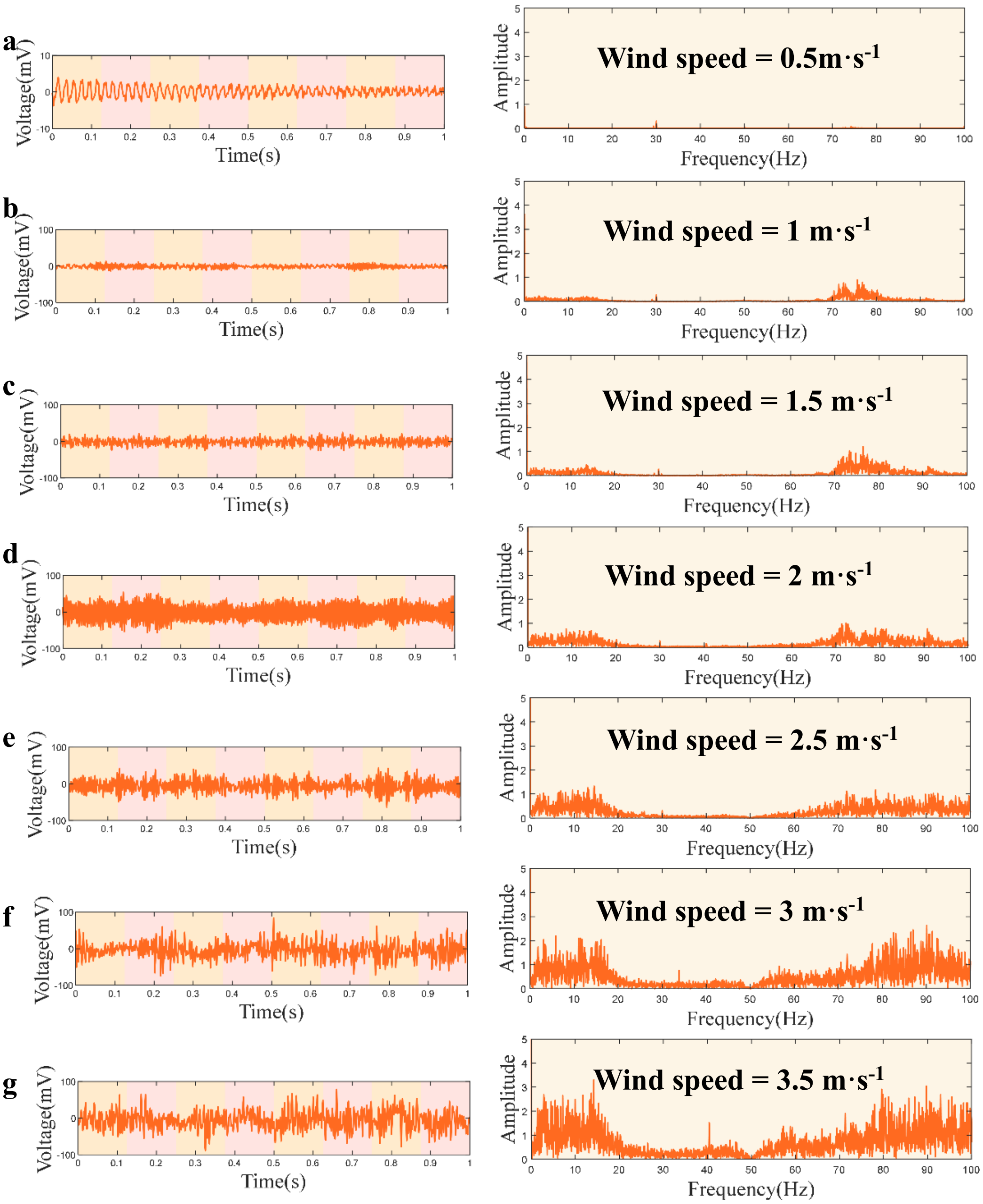

Supplementary Fig. 12: Voltage signals and spectrograms of a single feather at various wind speeds (without flapping).

Securing a single feather in the absence of flapping, signals were recorded under varying wind speeds ranging from 0.5 m·s^{-1} to 3.5 m·s^{-1} with an increment of 0.5 m·s^{-1}. Without flapping excitation, higher wind speeds led to more chaotic signals, resulting in a slight increase in signal amplitude. However, the amplitudes generally remained within ±100 mV. In comparison to the voltage signals induced by flapping excitation (Supplementary Fig. 11), the impact of ambient airflow-induced feather vibrations on

the amplitude of voltage signals was minimal.

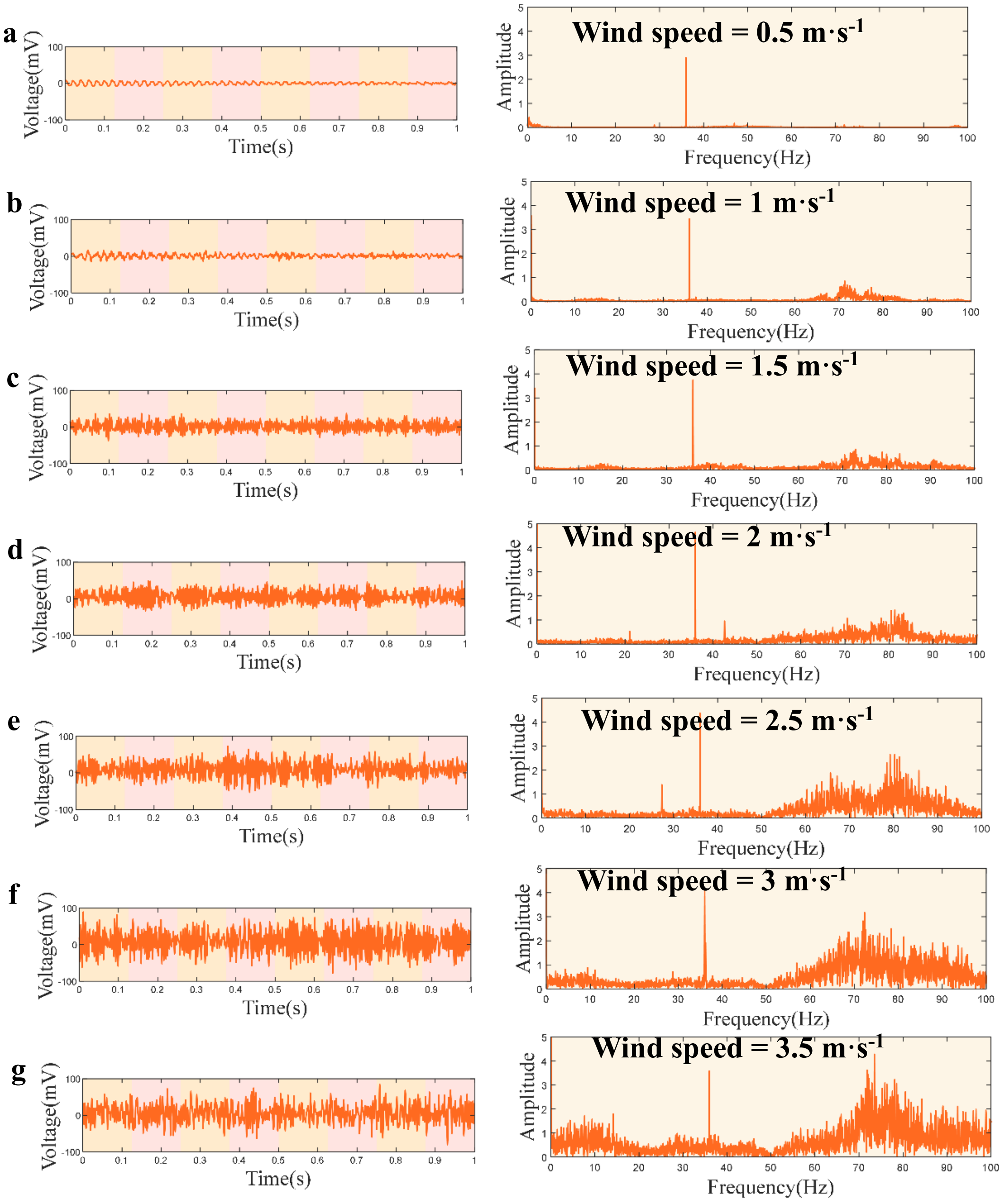

Supplementary Fig. 13: Voltage signals and spectrograms of multi-feather structures at various wind speeds (without flapping).

Securing multi-feather structures in a non-flapping state, signals were collected at various wind speeds ranging from 0.5 $m \cdot s^{-1}$ to 3.5 $m \cdot s^{-1}$ with an increment of 0.5 $m \cdot s^{-1}$. In comparison to voltage signals induced by flapping excitation (see Supplementary Fig. 14), the influence of incoming airflow on the amplitude of voltage signals caused by feather vibration is relatively minimal. Contrasting the signals under different wind

speeds for a single feather (see Supplementary Fig. 12), it can be observed that in the low-frequency range (0-20 Hz), the signal spectrum amplitude of a single feather is slightly larger than that of the multiple feather structure. However, in the high-frequency range (50-100 Hz), the signal spectrum amplitude of the multiple feather structure is overall slightly larger than that of a single feather, with more pronounced spectral peaks.

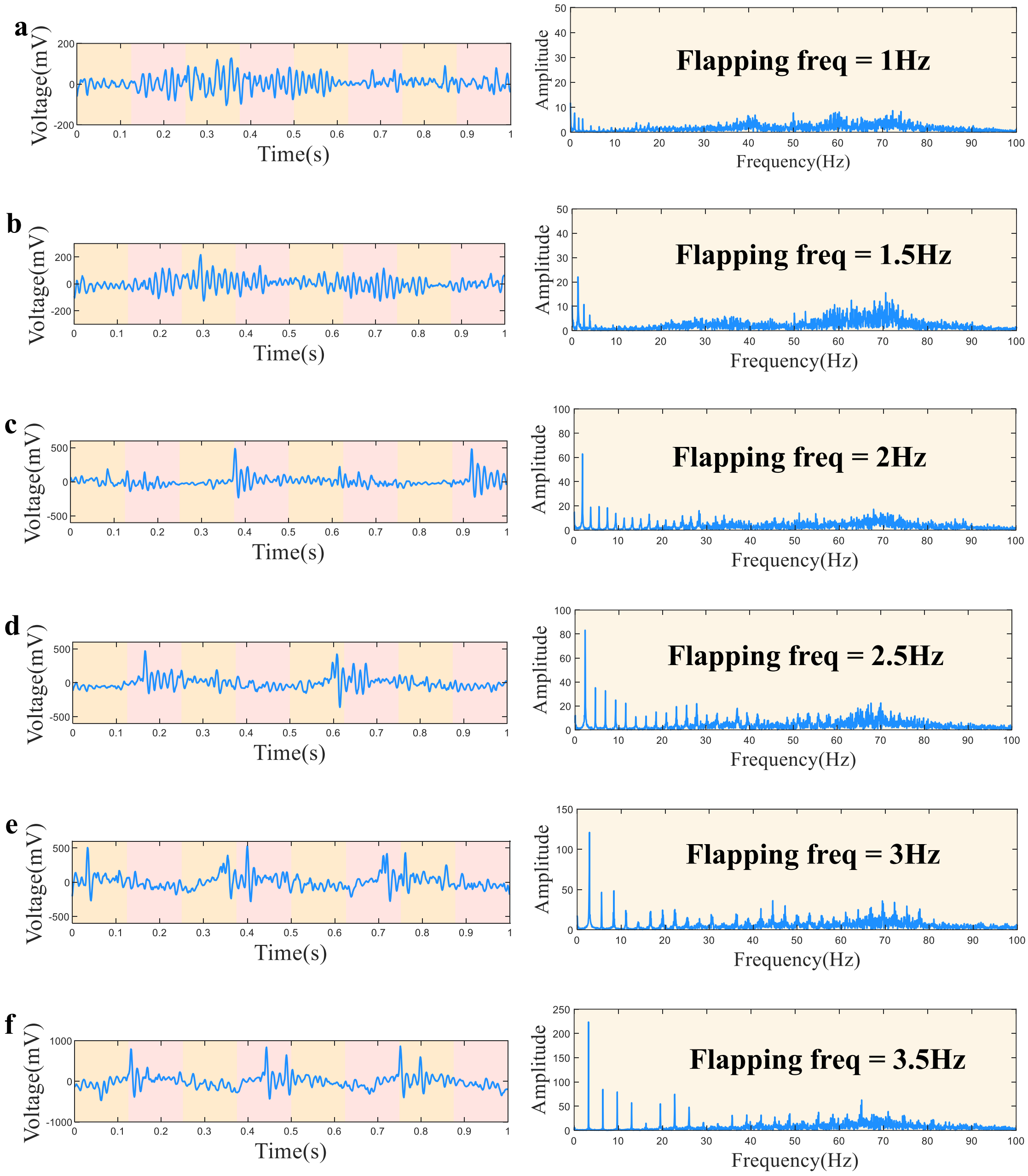

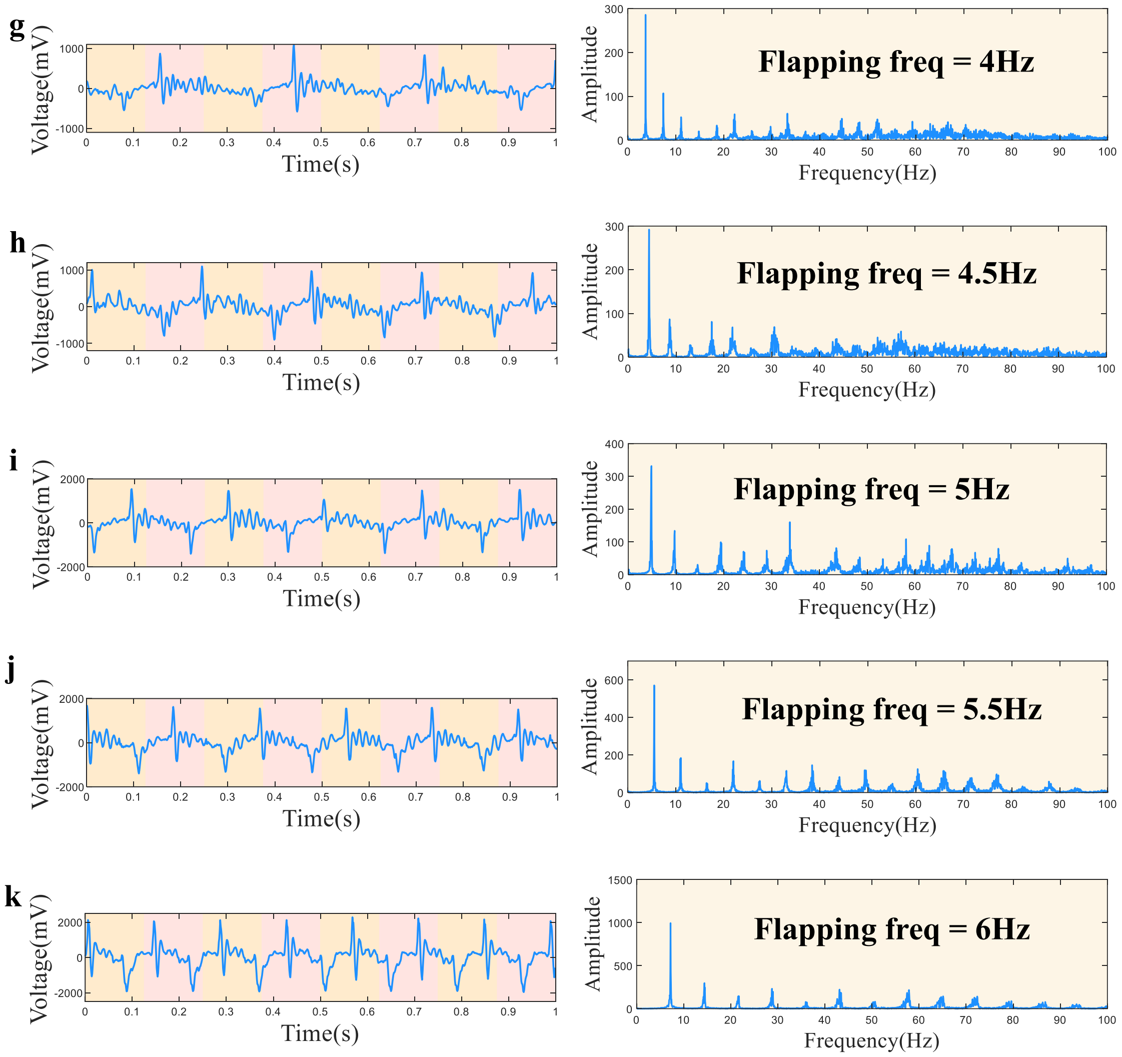

Supplementary Fig. 14: Voltage signals and spectrograms of multi-feather structures at various flapping frequencies.

Utilizing the multi-feather wing structure, flapping signals were collected on the flapping mechanism shown in Supplementary Fig. 6, with flapping frequencies ranging from 1 Hz to 6 Hz and an increment of 0.5 Hz. Fast Fourier Transform (FFT) was employed for spectral analysis. As the flapping frequency increased, the amplitude of the time-domain curve increased, maintaining a consistent trend with a single feather (see Supplementary Fig. 11). From the spectrogram, it can be observed that the signal frequencies are primarily dominated by the fundamental frequency and its harmonics. The amplitude of the fundamental frequency shows an increasing trend, with more pronounced noise concentrated in the frequency range of 60 Hz to 80 Hz. With the increase in flapping frequency, the noise in the high-frequency range gradually

diminishes.

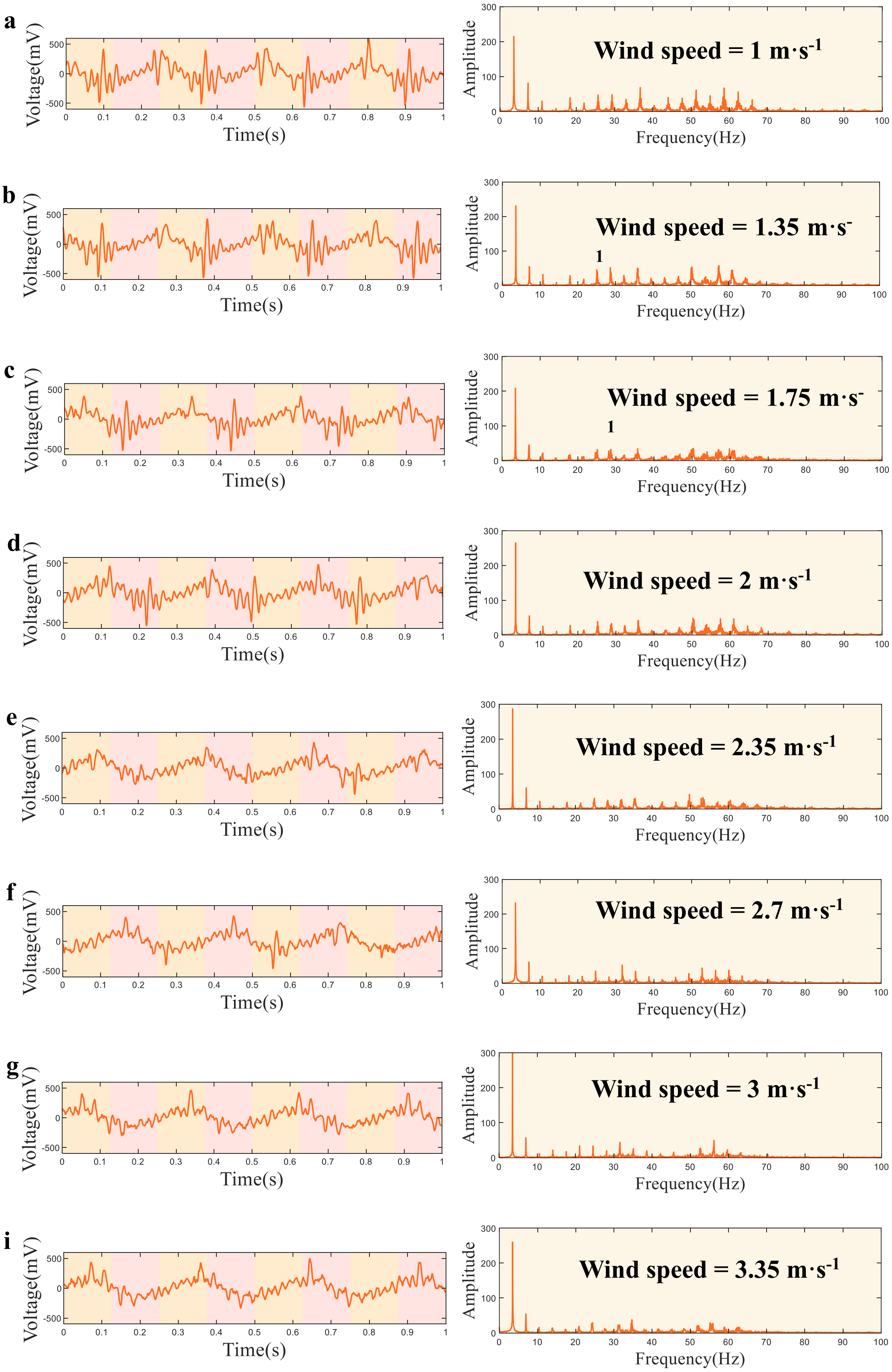
a
b
c
d
e
f
g
i
Voltage(mV)
Time(s)
Amplitude
Frequency(Hz)
Wind speed = 1 m·s-1
Wind speed = 1.35 m·s-1
Wind speed = 1.75 m·s-1
Wind speed = 2 m·s-1
Wind speed = 2.35 m·s-1
Wind speed = 2.7 m·s-1
Wind speed = 3 m·s-1
Wind speed = 3.35 m·s-1

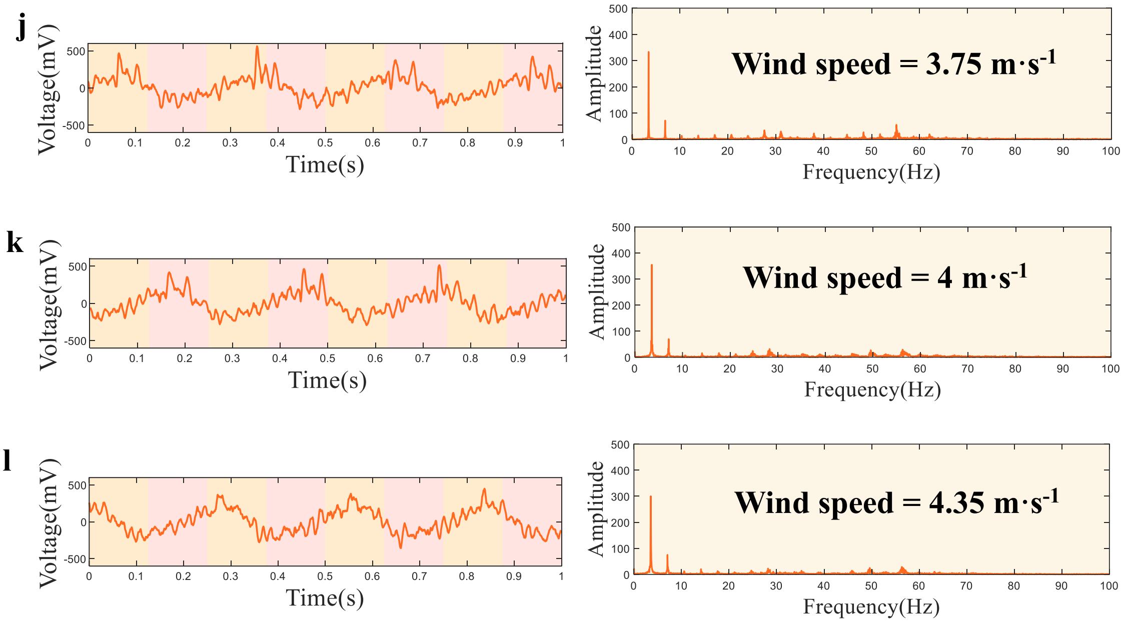

Supplementary Fig. 15: Voltage signals and spectrograms of multi-feather structures flapping at 3.5Hz under various wind speeds.

With the increase in wind speed, there is not a significant change in the amplitude of the time-domain curve and the amplitude of the fundamental frequency. The voltage signal induced by the flapping excitation remains dominant, while the variation in wind speed primarily affects the distribution characteristics of the noise.

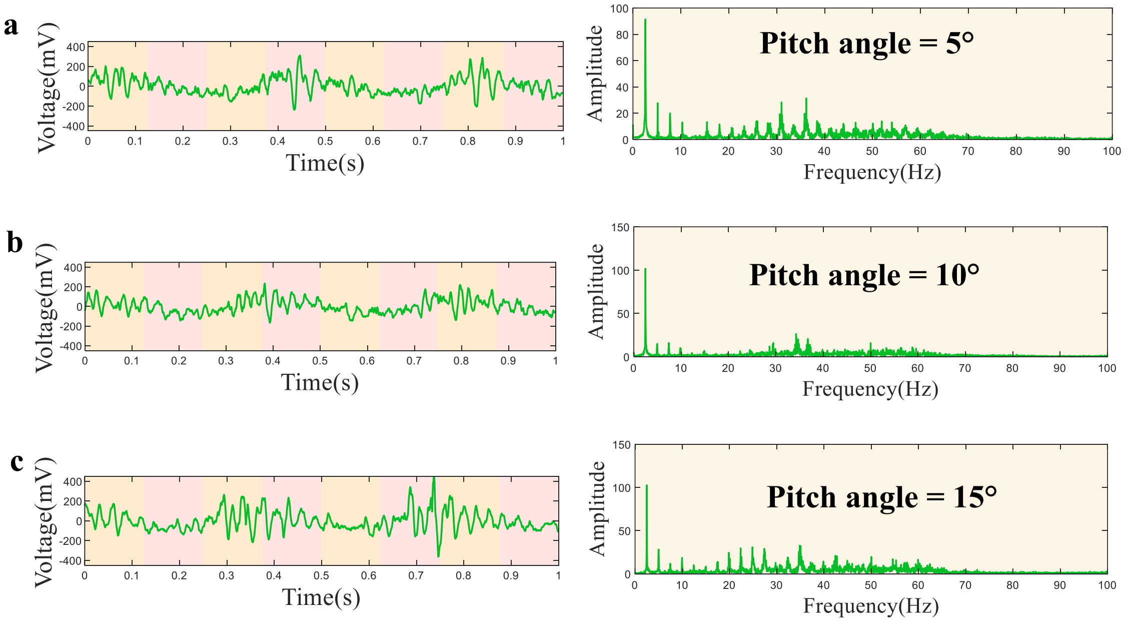

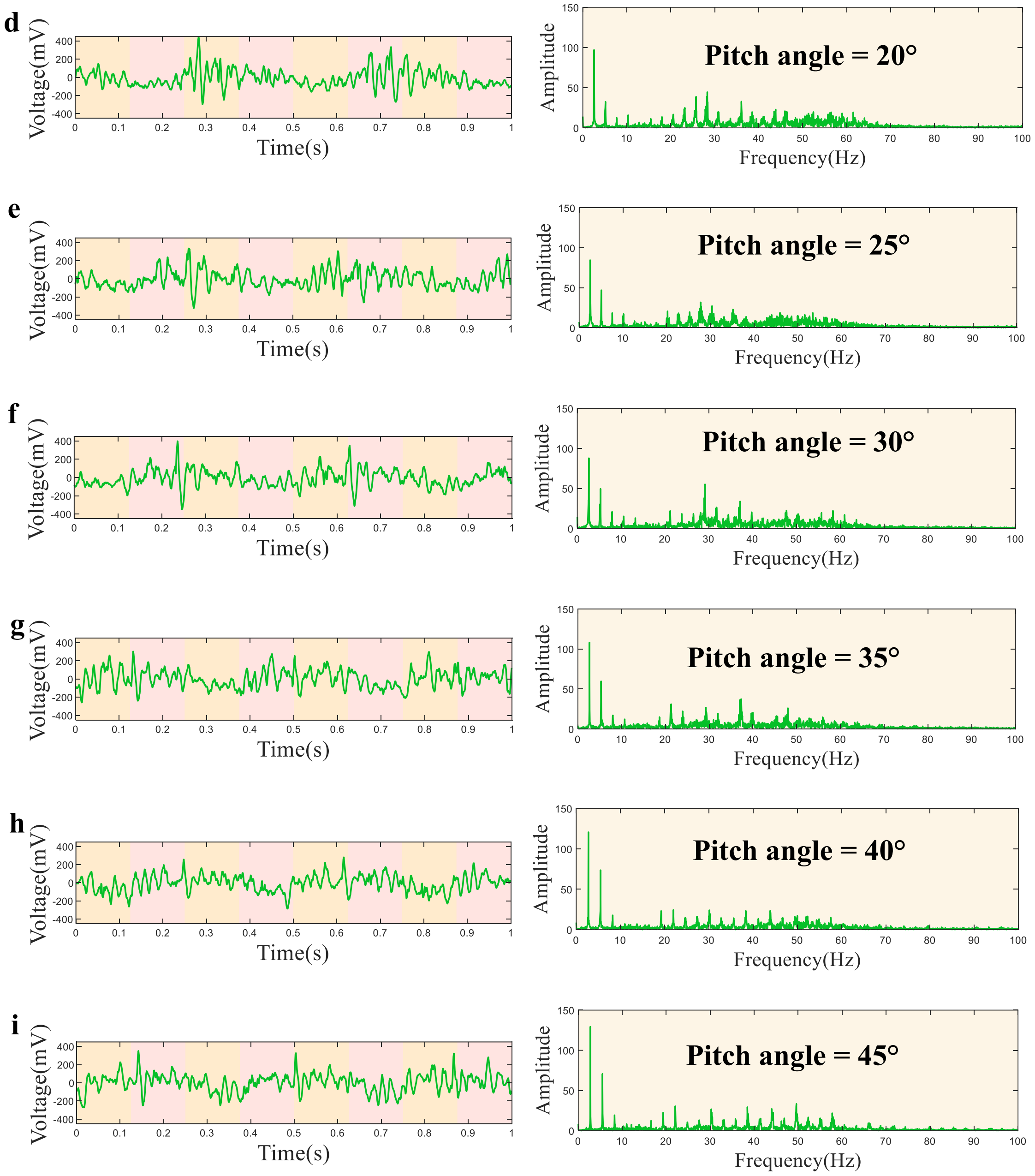

Supplementary Fig. 16: Voltage signals and spectrograms of multi-feather structures flapping at 2.5Hz with 3 $m \cdot s^{-1}$ wind speeds under various pitch angles.

7.Flapping wing kinematic and dynamic theoretical model

To quantitatively analyze the force characteristics generated by wing motion under different operating conditions, thereby further characterizing the signal differentiation brought about by PVDF force-electric conversion, we adopted the quasi-steady-state model proposed by Wang et al[7]. This model includes four aerodynamic force components: wing translation, rotation, their coupling, and added-mass effect. Building upon their work, we considered the influence of oncoming flow.

The model assumes that the fluid is incompressible, with fluid density treated as a constant. The wings are assumed to be rigid, and the wings made of feathers have relatively high stiffness, which is reasonable. Throughout the stroke, the aerodynamic forces acting on the wing are perpendicular to the chord. It is assumed that, during an infinitesimally small duration, the transient loads in wing flapping are equal to the steady-state loads at the same instantaneous translational velocity, angular velocity, and pitch angle.

We established a physical model as shown in Supplementary Fig. 17, taking into account the variation of velocity and acceleration along the wing span. The model employs the Blade Element Method (BEM) to discretize the wing into finite-width chordwise strips. The total load generated can be calculated by integrating the loads on all strips over the entire wing

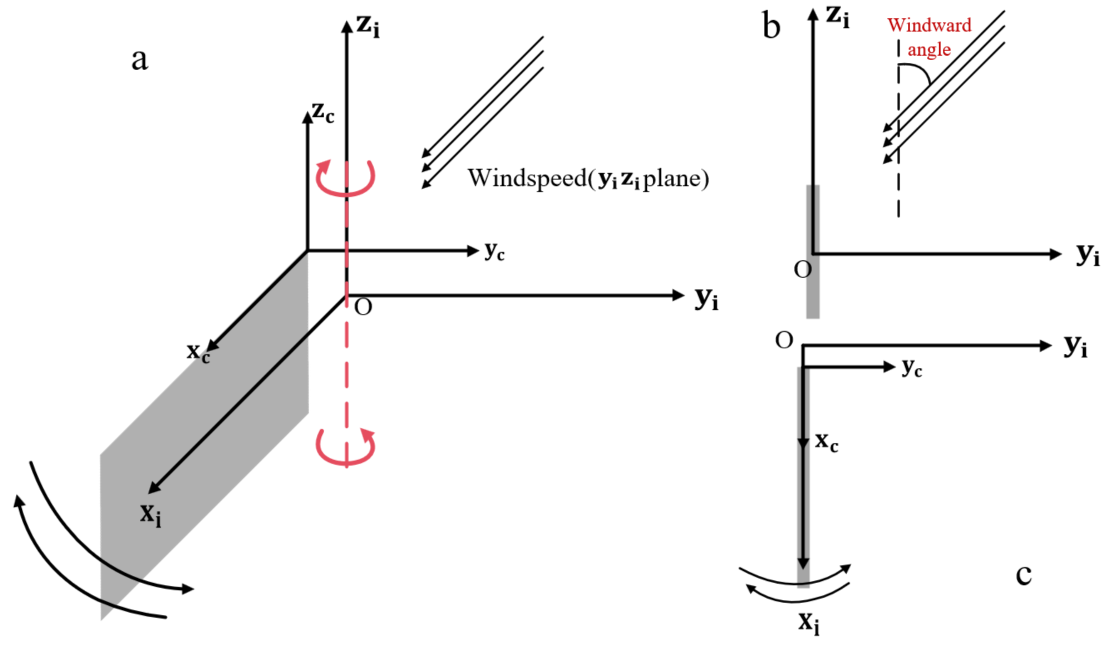

Supplementary Fig. 17: Flapping wing kinematic model. a Flapping motion in the three-dimensional inertial coordinate system and the co-rotating coordinate system, with the wing (in gray) flapping about the $\mathbf{z_i}$ axis (red line). **b** Observation view along the $-\mathbf{x_i}$ direction, where the oncoming flow velocity is in the $\mathbf{y_i z_i}$ plane. **c** Observation view along the $-\mathbf{z_i}$ direction.

7.1 Kinematics

We quantify the motion of a rigid flapping wing through three Euler angles: the sweep angle φ, the pitch angle θ, and the yaw angle η. Simultaneously, we define the inertial coordinate system $\mathbf{x_i y_i z_i}$ fixed to the ground and the co-rotating coordinate system $\mathrm{x_c y_c z_c}$ moving with the wing, where the $\mathbf{x_c}$ axis coincides with the pitch axis. Using these three Euler angles, we can represent three consecutive rotational motions (sweeping motion, pitching motion, and yawing motion):

$$R_{\varphi} = \begin{bmatrix} \cos\varphi & -\sin\varphi & 0 \\ \sin\varphi & \cos\varphi & 0 \\ 0 & 0 & 1 \end{bmatrix}, R_{\theta} = \begin{bmatrix} \cos\theta & 0 & \sin\theta \\ 0 & 1 & 0 \\ -\sin\theta & 0 & \cos\theta \end{bmatrix}, R_{\eta} = \begin{bmatrix} 1 & 0 & 0 \\ 0 & \cos\eta & -\sin\eta \\ 0 & \sin\eta & \cos\eta \end{bmatrix} \tag{1}$$

The angular velocity $\boldsymbol{\omega}_c$ and angular acceleration $\boldsymbol{\alpha}_c$ in the attached coordinate system can be expressed as:

$$\boldsymbol{\omega}_c = \begin{bmatrix} \dot{\eta} - \dot{\varphi}\sin\theta \\ \dot{\theta}\cos\eta + \dot{\varphi}\cos\theta\sin\eta \\ \dot{\varphi}\cos\eta\cos\theta - \dot{\theta}\sin\eta \end{bmatrix} \tag{2}$$

$$\boldsymbol{\alpha}_\mathbf{c} = \dot{\boldsymbol{\omega}}_c \begin{bmatrix} \ddot{\eta} - \ddot{\phi}\sin\theta - \dot{\phi}\dot{\theta}\cos\theta \\ \ddot{\phi}\cos\theta\sin\eta + \ddot{\theta}\cos\eta - \dot{\eta}\dot{\theta}\sin\eta + \dot{\phi}\left(\dot{\eta}\cos\eta\cos\theta - \dot{\theta}\sin\eta\sin\theta\right) \\ \ddot{\phi}\cos\eta\cos\theta - \ddot{\theta}\sin\eta - \dot{\eta}\dot{\theta}\cos\eta - \dot{\phi}\left(\dot{\eta}\cos\theta\sin\eta + \dot{\theta}\cos\eta\sin\theta\right) \end{bmatrix} \tag{3}$$

In the co-rotating coordinate system, the translational velocity and acceleration of a point with the position vector $\mathbf{r} = [x_c, 0, 0]^T$ along the pitch axis can be calculated using the equation:

$$\mathbf{v}_c = \boldsymbol{\omega}_c \times \mathbf{r} = x_c\left[0, \omega_{z_c} - \omega_{y_c}\right]^T \tag{4}$$

$$\mathbf{a}_\mathbf{c} = \boldsymbol{\alpha}_\mathbf{c} \times \mathbf{r} + \boldsymbol{\omega}_\mathbf{c} \times \mathbf{v}_\mathbf{c} = x_c\left[-\omega_{y_c}^2 - \omega_{z_c}^2, \alpha_{z_c} + \omega_{x_c}\omega_{y_c}, \omega_{x_c}\omega_{z_c} - \alpha_{y_c}\right]^T \tag{5}$$

When considering the influence of the incoming flow, it is necessary to vectorially sum the flow velocity with $\mathbf{v}_\mathbf{c}$.

7.2 Translation-induced load

Leading-edge vortex (LEV) effects dominate the force generation in translational wings, and according to experimental results, the lift coefficient can be approximately expressed as:

$$C_L^{\text{trans}} = A\sin(2\alpha) \tag{6}$$

where α is defined as the angle of attack of the rigid wing:

$$\alpha = \arctan\left(\left|\frac{v_{y_c}}{v_{z_c}}\right|\right) \tag{7}$$

Define A_R as the aspect ratio, where A is a constant coefficient, taken as:

$$A = \frac{\pi \text{A\_R}}{2 + \sqrt{\text{A\_R}^2 + 4}} \tag{8}$$

In this quasi-steady model, the translational lift coefficient C_L^{trans} is directly utilized for evaluating translational aerodynamic forces. Under the assumption that the force is perpendicular to the wing surface, the translational drag coefficient and the total force coefficient are defined as follows:

$$C_D^{\text{trans}} = C_L^{\text{trans}}\tan(\alpha) \tag{9}$$

$$C_{Fy_c}^{\text{trans}} = C_L^{\text{trans}}/\cos(\alpha) \tag{10}$$

The induced translational force $F_{y_C}^{\text{trans}}$ generated by this can be calculated through the wing surface integration:

$$F_{y_C}^{\text{trans}} = \int_0^R -\operatorname{sgn}(v_{y_c})\frac{1}{2}\rho_f v_c^2 C_{Fy_c}^{\text{trans}}\, c\, dx_c \tag{11}$$

where c is the chord length, and $\operatorname{sgn}(\cdot)$ denotes the sign function.

7.3 Rotation-induced load

When the wing rotates about any axis in the medium, it experiences distributed loads. For a rotating wing, the chordwise direction induces different velocities, with the amplitude linearly increasing with the distance to the pitch axis. Therefore, the induced rotational force can be calculated by integrating the load over the entire wing surface:

$$F_{y_c}^{\text{rot}} = \int_0^R \int_{\hat{d}c-c}^{\hat{d}c} \frac{\rho_f}{2}\omega_{x_c}\left|\omega_{x_c}\right| C_D^{\text{rot}} z_c |z_c|\, dz_c\, dx_c \tag{12}$$

where C_D^{rot} is the rotational drag coefficient, $\hat{d}c - c$ and $\hat{d}c$ represent the coordinates of the TE and LE of the wing in the $\mathbf{z_c}$ direction. When the angle of attack is 90°, using the translational drag coefficient C_L^{trans} from the pitching motion as the rotational drag coefficient is more accurate, i.e.:

$$C_D^{\text{rot}} = C_D^{\text{trans}}\left(\alpha = \frac{\pi}{2}\right) = \frac{2\pi A\_R}{2 + \sqrt{A\_R^2 + 4}} \tag{13}$$

7.4 Coupling load

Due to the nonlinearity introduced by the interaction of the flow and the wing, a wing undergoing both translational and rotational motion generates additional forces. These additional forces can be explained by the coupling effects between the translational and rotational motion of the wing. The loads caused by coupling effects consist of two load components, as follows:

$$F_{y_c}^{\text{coup}} = \begin{cases} \int_0^R -\pi\rho_f\omega_{x_c}v_{z_c}\left[\left(\frac{3}{4}-\hat{d}\right)c^2+\frac{1}{4}c^2\right]dx_c \ , v_{z_c}\geq 0 \\ \int_0^R -\pi\rho_f\omega_{x_c}v_{z_c}\left[\left(\hat{d}-\frac{1}{4}\right)c^2+\frac{1}{4}c^2\right]dx_c \ , v_{z_c}\leq 0 \end{cases} \quad (14)$$

7.5 Added-mass load

During wing flapping, the surrounding fluid accelerates or decelerates in response to wing motion, with this effect being most pronounced during the stroke reversal phase. Simultaneously, the accelerating fluid exerts a reactive force on the flapping wings. This interaction can be simulated by an added mass coefficient multiplied by the wing acceleration in the direction opposite to the flapping motion. For a chordwise strip with chord length c, unit width, and normalized offset d from the leading edge with respect to the pitching axis, the added mass coefficient matrix is given by:

$$M = \begin{bmatrix} m_{22} & m_{24} \\ m_{42} & m_{44} \end{bmatrix} = \frac{\pi}{4}\rho_f c^2 \begin{bmatrix} 1 & c\left(\frac{1}{2}-\hat{d}_0\right) \\ c\left(\frac{1}{2}-\hat{d}_0\right) & \frac{1}{32}c^2 + c^2\left(\frac{1}{2}-\hat{d}_0\right)^2 \end{bmatrix} \quad (15)$$

The loads induced by the added mass effect are given by:

$$[F_{y_c}^{\text{am}}, \tau_{x_c}^{\text{am}}]^T = -\int_0^R M\left[a_{y_c}, \alpha_{x_c}\right] dx_C \quad (16)$$

Here, a_{y_c} is the translational acceleration in the y_c direction, and α_{x_c} is the rotational acceleration in the x_c direction. Thus, the aerodynamic forces calculated by this quasi-steady theory are given by:

$$F_{y_c}^{a} = F_{y_c}^{\text{trans}} + F_{y_c}^{\text{rot}} + F_{y_c}^{\text{coup}} + F_{y_c}^{\text{am}} \quad (17)$$

We implemented corresponding programs using MATLAB software to simulate flapping wing motion. The wingspan is 29 cm, chord length is 7 cm, and the flapping angle φ is given by: where φ_m represents the flapping magnitude with a value of 40°, and f is the flapping frequency.

$$\varphi = \varphi_m \sin(2\pi f t) \quad (18)$$

where φ_m represents the flapping magnitude with a value of 40°, and f is the flapping frequency.

We conducted simulations to calculate the aerodynamic forces acting on a single wing under different flapping frequencies (0-6 Hz), freestream velocities (0-3 m·s^{-1}), and pitch angles (0-30°). Here, lift is defined as the force in the y-axis direction, and Lateral force is defined as the force in the x-axis direction.

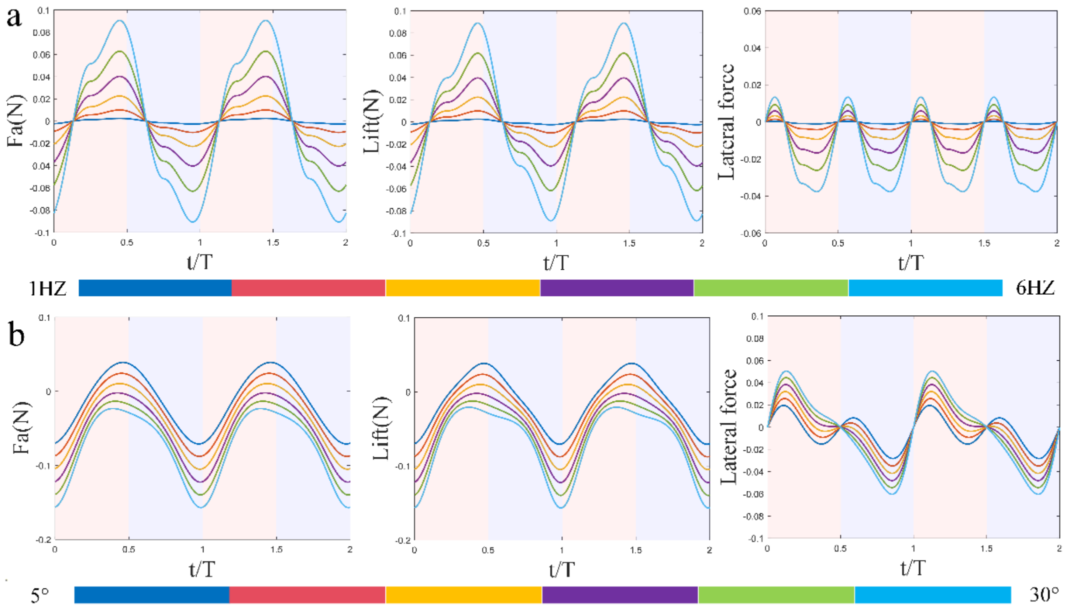

Supplementary Fig. 18: Simulation of load magnitude on a single wing under various operating conditions. a Under flapping at frequencies of 1Hz to 6Hz with a body pitch angle of 0° and zero inflow velocity, the total force, lift, and lateral force acting on the wing are evaluated. **b** With a flapping frequency of 3Hz, an inflow velocity of 3 m·s^{-1}, and body pitch angles ranging from 5° to 30°, the total force, lift, and lateral force on the wing are evaluated. Source data are provided as a Source Data file.

Observing Supplementary Fig. 18a, it can be noted that with the increase in flapping frequency, the aerodynamic total force $\mathbf{F}_a$ and lift amplitude on a single wing both increase, exhibiting clear periodicity and growth patterns. The force $\mathbf{F}_a$ and lift show symmetry in the upstroke and downstroke, while the lateral force exhibits asymmetry due to the wing's asymmetry along the x-axis. The force along the z-axis remains zero. Examining Supplementary Fig. 18b, with the increase in body pitch angle,

the aerodynamic total force $\mathbf{F}_a$ and lift time history curve maintain periodicity, and a quasi-translational shift occurs, while the peak-to-peak value remains largely unchanged. However, the amplitude of the lateral force increases. Through numerical simulations, it is observed that under different conditions, the aerodynamic force curves on the wing exhibit distinct characteristics. This lays the theoretical foundation for our exploration of wing-flapping motion perception and recognition.

8.Nonlinear analysis

Aircraft and vehicle lighting engineering systems are often subjected to various excitations, inevitably resulting in a variety of vibrations. The majority of these vibrations belong to nonlinear vibrations. In comparison to linear systems, they generate more complex vibrational responses, profoundly affecting fields such as aircraft structural vibration, fatigue analysis, and signal acquisition.

Taking a linear single-degree-of-freedom system as an example, when subjected to periodic excitation forces, its dynamic equation is given by:

$$m\ddot{x} + c\dot{x} + kx = F_0 \cos{(\omega t)} \tag{1}$$

We set the coefficients as follows: $m = 1; c = 0.01; k = 1; F_0 = 3; \omega = 1.25$. Through MATLAB programming, we calculate the time-history curve $x(t)$. After a long duration, we employ the Fast Fourier Transform (FFT) algorithm to obtain the corresponding frequency spectrum, as illustrated in Supplementary Fig. 19.

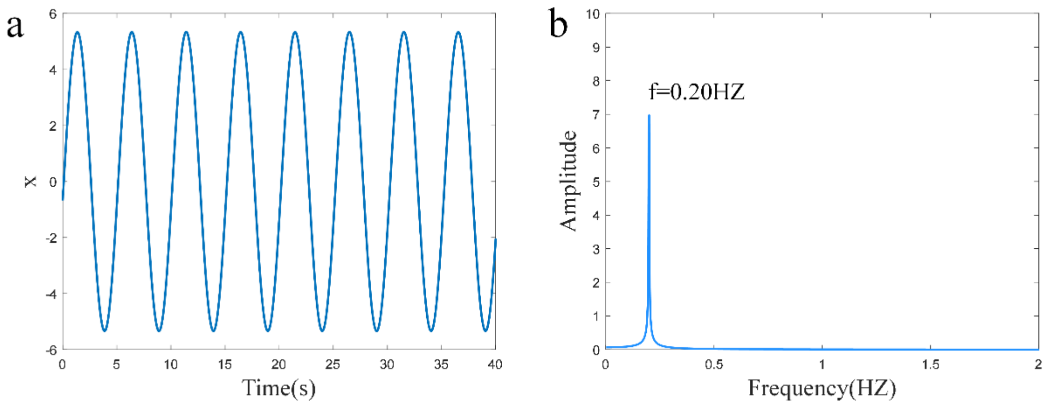

Supplementary Fig. 19: Forced vibration response of the linear system. a Time-history curve. **b** Frequency spectrum.

In a linear single-degree-of-freedom system, significant resonance occurs when the excitation force frequency is close to the natural frequency of the system, while other excitation force frequencies do not induce resonance. However, in a nonlinear single-degree-of-freedom system, besides the primary resonance, significant vibrations can also occur near other frequencies.

When introducing the cubic nonlinearity term [8]:

$$m\ddot{x} + c\dot{x} + kx + \beta x^3 = F_0 \cos(\omega t) \tag{2}$$

We set the coefficients as follows: $m = 1; c = 0.01; k = 1; F_0 = 3; \omega = 1.25; \beta = 0.2$. The resulting time-history curve and frequency spectrum are shown in Supplementary Fig. 20, revealing the generation of a triple frequency component in response to the external excitation force.

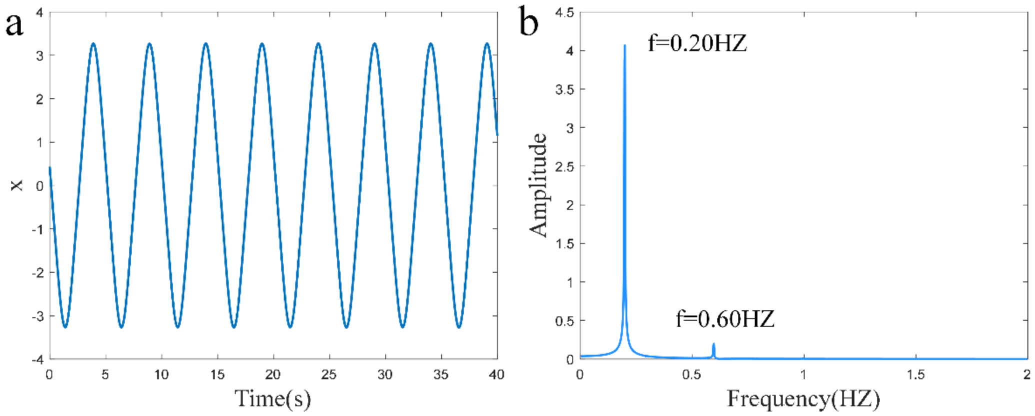

Supplementary Fig. 20: Forced vibrational response of systems with cubic nonlinearities. a Time-history curve. **b** Frequency spectrum.

When introducing the quadratic nonlinear term [8]:

$$m\ddot{x} + c\dot{x} + kx + \beta x^2 = F_0 \cos(\omega t) \quad (3)$$

With coefficients set as $m = 1; c = 0.01; k = 1; F_0 = 3; \omega = 1.25; \beta = 0.1$, the time-history curve and frequency spectrum are observed, as shown in Supplementary Fig. 21, revealing the appearance of the external excitation frequency's second harmonic in the response.

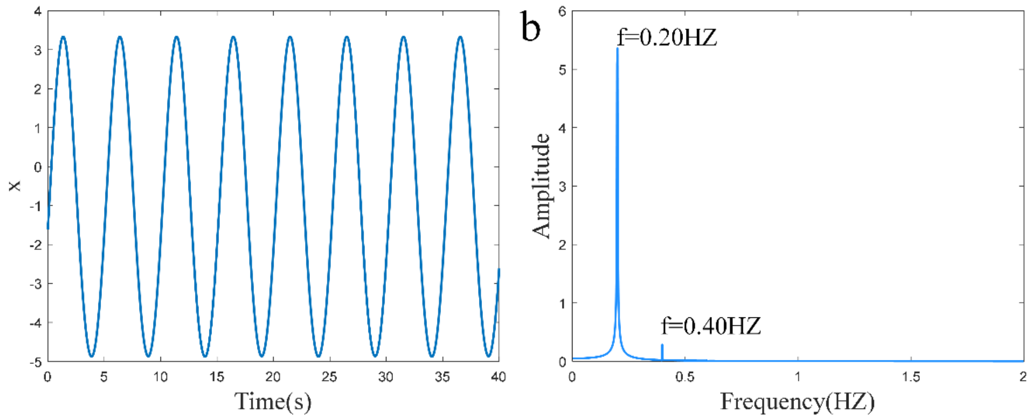

Supplementary Fig. 21: Forced vibrational response of systems with quadratic nonlinearities. a Time-history curve. **b** Frequency spectrum.

When both the square and cubic nonlinear terms are present [8] :

$$m\ddot{x} + c\dot{x} + kx + \beta_1 x^2 + \beta_2 x^3 = F_0 \cos(\omega t) \quad (4)$$

With coefficients set as $m = 1; c = 0.01; k = 1; F_0 = 3; \omega = 1.25; \beta_1 = 0.3; \beta_2 = 0.2$, the time- history curve and frequency spectrum are observed, as shown in Supplementary Fig. 22, indicating the simultaneous occurrence of the external excitation frequency's second and third harmonics in the response.

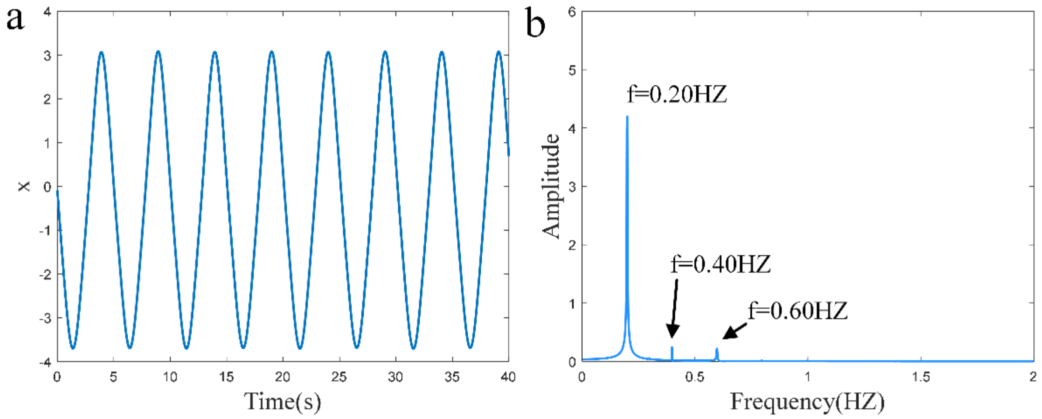

Supplementary Fig. 22: Response of systems with second and third-order nonlinearities to forced vibrations. a Time-history curve. **b** Frequency spectrum.

With coefficients set as $m = 1; c = 0.01; k = 1; F_0 = 3; \omega = 1; \beta_1 = 1; \beta_2 = 1$, the time-history curve and frequency spectrum are observed, as shown in Supplementary Fig. 23, revealing the simultaneous generation of the external excitation frequency's second, third, fourth, and fifth harmonics in the response. At this point, the system's natural frequency equals the excitation frequency, and the system experiences resonance, generating multiple harmonic frequencies.

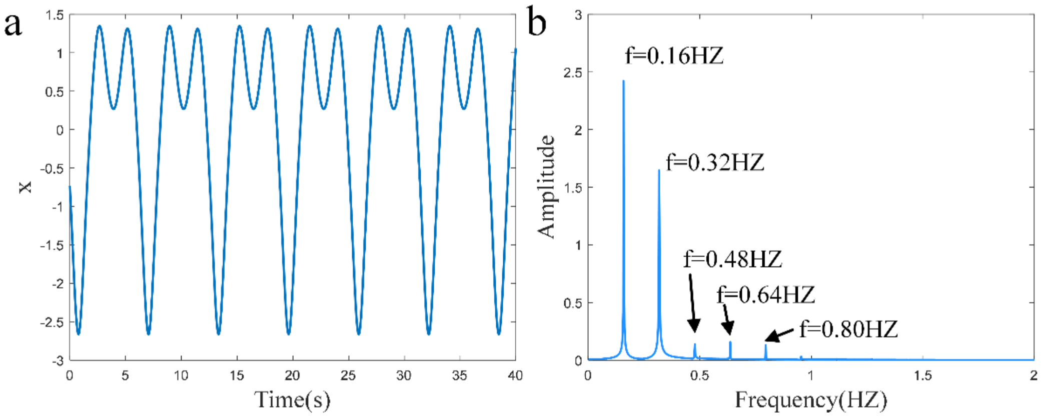

Supplementary Fig. 23: Resonance response of systems with second and third-order nonlinearities. a Time-history curve. **b** Frequency spectrum.

Therefore, the introduction of nonlinear terms into the vibrational system results in a complex array of vibration frequency components. For feathers, the inherent multi-barb structure and collisions among the feathers' arranged structures introduce nonlinearity into the system. The incorporation of these nonlinear effects [8] can provide a theoretically explanatory basis for the generation of complex and distinct voltage signals during the flapping process. The signals produced by our perception structural unit during flapping motion exhibit prominent harmonic frequencies (Supplementary Fig. 11-16), which is consistent with the analysis results presented above.

9.The convolutional neural network-grey wolf optimization

To determine the optimal input sample size, we used a series of input sample sizes from 0.01 s to 10 seconds to investigate the effect of sample size on recognition accuracy. We calculate the root mean square error in flapping frequency identification by the Convolutional Neural Network (CNN) under different input sample sizes, in which we conducted 5 network training sessions for each type of input sample size, and took the average of the five root mean square errors as the final root mean square error as shown in the Supplementary Fig. 24 below to ensure the reliability of the results. The findings indicate that as the input sample size decreases, the recognition error gradually increases. when the input sample size is 0.01s, the recognition error approaches 0.9 Hz. Conversely, when the input sample size increases to 1 second, the recognition error decreases to 0.1 Hz. Furthermore, as the sample size continues to increase, the recognition error gradually tends to be stable, and the recognition accuracy plateaus. Consequently, to balance recognition accuracy with the complexity of network training, we choose 1 second as the size of the input sample.

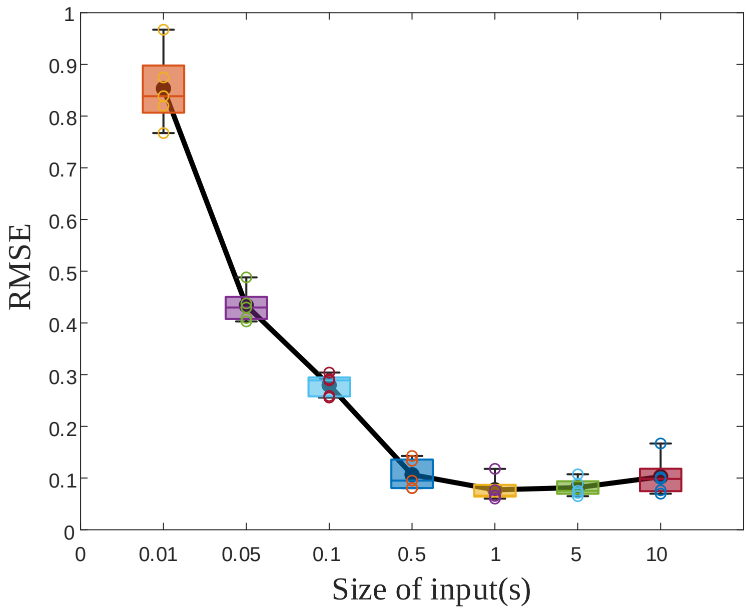

Supplementary Fig. 24: Root mean square error (RMSE) in flapping frequency identification across diverse sample input dimensions. The box plots show the maximum, minimum, 25th percentile, 75th percentile, and median error values for each category. Black points represent the mean error for each size, the colored circles

represent the data distribution (sample number of each size n = 5). Source data are provided as a Source Data file.

We performed data segmentation for the voltage signals generated by the feather-PVDF smart material under different flapping frequencies, wind speeds, and pitch angles. Each sample had a length of 1s, and all samples were randomly split into three sets: training set (80%), testing set (10%), and validation set (10%) for input into the network for training and testing. The initial data were normalized and transformed into the input format of 1000×1×1×N (N is the number of samples) for training. The network structure is illustrated in Fig. 4g, comprising three convolutional layers, three pooling layers, and three fully connected layers, with the specific stacking sequence as follows: data input → convolutional layer → loss function (ReLU) → pooling layer → convolutional layer → loss function (ReLU)→ pooling layer → convolutional layer → loss function (ReLU) → pooling layer → fully connected layers (3 layers) → regression layer. The root mean square error (RMSE) (1) and mean absolute percentage error (MAPE) (2) between the fitted results and true labels were used as performance metrics to evaluate the fitting accuracy. RMSE was also used as the fitness function for the Grey Wolf Optimizer (GWO). In this process, GWO aimed to minimize the fitness value to determine the optimization direction of key parameters. After 100 iterations, the final critical network hyperparameters were obtained, as shown in Supplementary Tables 1-3. The change in fitness function value over iterations is depicted in Supplementary Fig. 25. The final optimized network was used for training, and the change in the loss function over iterations is shown in Supplementary Fig. 25. The network demonstrated good stability during longer iteration periods, indicating robust training of the recognition network. The fitting results are illustrated in Fig. 4h, and the error histogram is presented in Supplementary Fig. 26. Errors were primarily concentrated around 0, indicating the feasibility of the recognition.

$$\mathrm{RMSE} = \sqrt{\frac{1}{N}\sum_{i=1}^{n}\left(Y_i - f(x_i)\right)^2} \quad (1)$$

$$\text{MAPE} = \frac{100\%}{n}\sum_{i=1}^{n}\left|\frac{\hat{y}_i - y_i}{y_i}\right| \tag{2}$$

Supplementary Table 1. The hyperparameters of the neural network for flapping frequency recognition

No	Layer Type	Kernel size	Kernels number	Pool Size	Stride	FC Size	Learning rate
1	Conv_1	206	143				
2	Maxpool_1			9	25		
3	Conv_2	231	38				
4	Maxpool_2			10	2		
5	Conv_1	39	24				
6	Maxpool_1			1	1		0.0017
7	FC					44	
8	FC					17	
9	FC					1	
10	Regression						

Supplementary Table 2. The hyperparameters of the neural network for oncoming flow velocity recognition

No	Layer Type	Kernel size	Kernels number	Pool Size	Stride	FC Size	Learning rate
1	Conv_1	184	197				
2	Maxpool_1			82	16		
3	Conv_2	148	159				
4	Maxpool_2			13	6		6.11e-04
5	Conv_1	1	206				
6	Maxpool_1			1	32		

7	FC					12	
8	FC					16	
9	FC					1	
10	Regression						

Supplementary Table 3. The hyperparameters of the neural network for pitch angle recognition

No	Layer Type	Kernel size	Kernels number	Pool Size	Stride	FC Size	Learning rate
1	Conv_1	145	188				0.0016
2	Maxpool_1			97	13		
3	Conv_2	66	224				
4	Maxpool_2			21	8		
5	Conv_1	48	143				
6	Maxpool_1			2	6		
7	FC					43	
8	FC					45	
9	FC					1	
10	Regression						

The optimization process of Grey Wolf Optimization (GWO) for a one-dimensional deep CNN is outlined as follows:

Step 1: Prepare a 1D deep CNN and set the root mean square error (RMSE) as the fitness function for GWO.

Step 2: Initialize the optimization hyperparameter vector $\mathbf{X}_i = [x_{1i}, x_{2i}, \cdots x_{ni}]$, determining the optimization range.

Step 3: Initialize the parameters of the Grey Wolf Optimizer, and train the 1D deep CNN network.

Step 4: Evaluate the fitness of each agent and identify the top three agents: $\mathbf{X}_\alpha$, $\mathbf{X}_\beta$

and $\mathbf{X}_\delta$.

Step 5: Update the position of each search agent.

Step 6: Update GWO parameters and return to Step 4, updating the top three agents ($\mathbf{X}_\alpha$, $\mathbf{X}_\beta$ and $\mathbf{X}_\delta$) until reaching the maximum iteration count.

Step 7: Return $\mathbf{X}_\alpha$ as the optimized parameter vector for the 1D deep CNN.

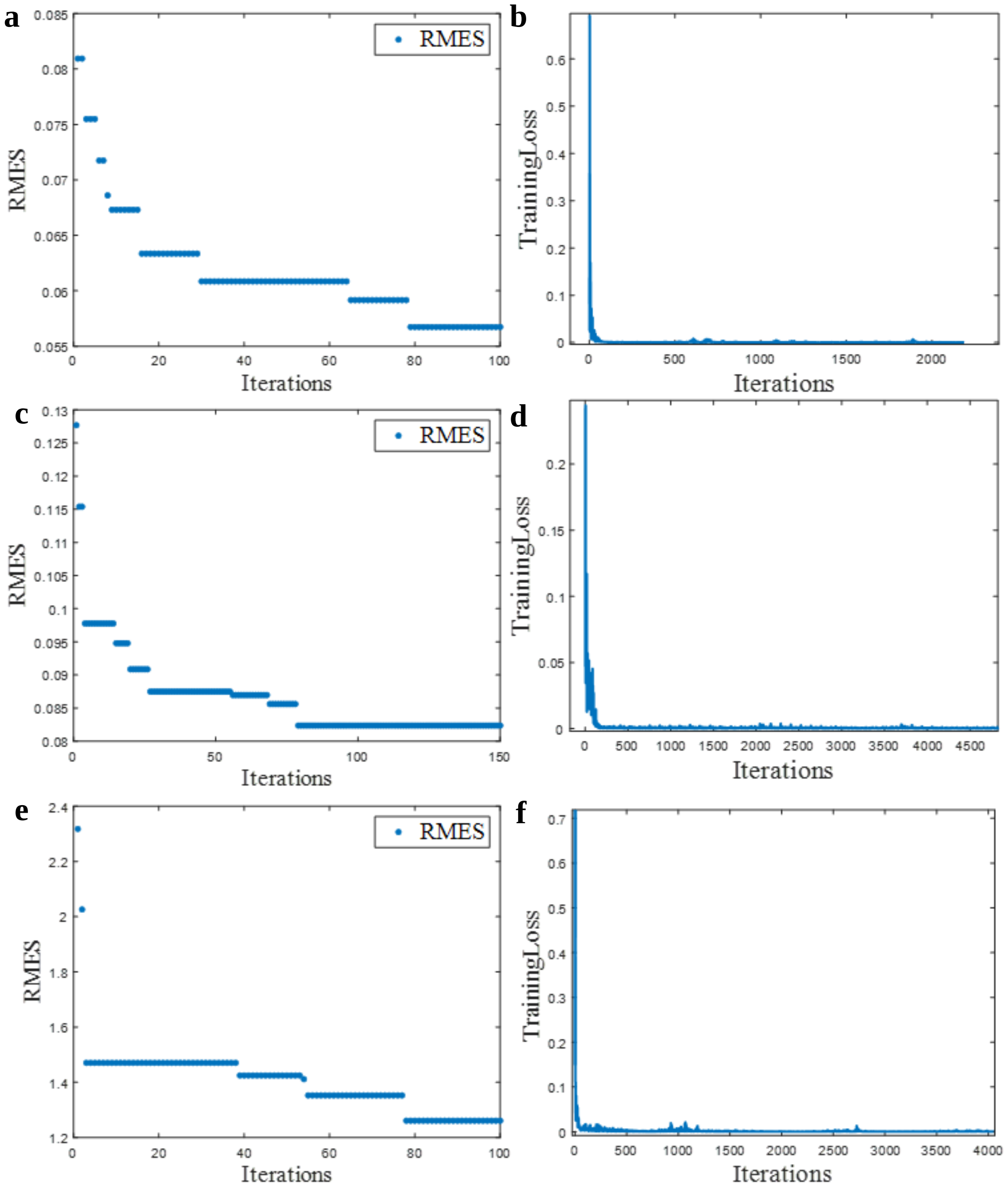

Supplementary Fig. 25: Fitness iteration graph of the grey wolf optimizer and iteration graph of the optimization network training loss function. a Iteration plot of RMSE for flapping frequency recognition in grey wolf optimization. **b** Training error iteration plot for flapping frequency recognition. **c** Iteration plot of RMSE for wind speed recognition in grey wolf optimization. **d** Training error iteration plot for wind

speed recognition. **e** Iteration plot of RMSE for pitch angle recognition in grey wolf optimization. **f** Training error iteration plot for pitch angle recognition.

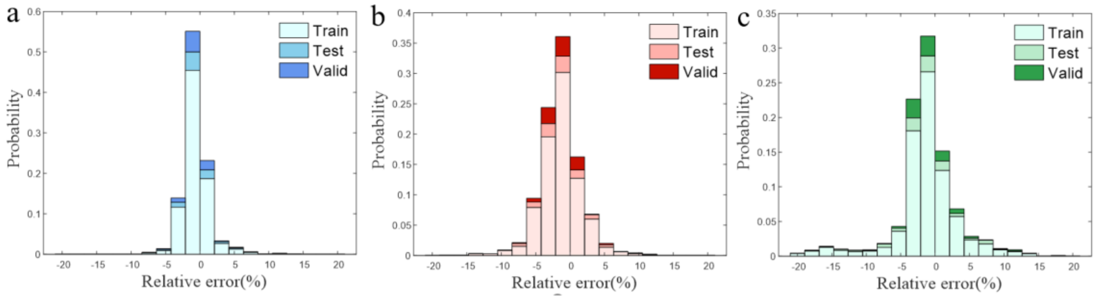

Supplementary Fig. 26: Histograms of convolutional neural network training errors. a Flapping frequency recognition. **b** Wind speed recognition. **c** Pitch angle recognition.

Supplementary Table 4. Comparative analysis of parameter identification error in our work versus other fixed-wing studies

Sensor type	Sensor num	Velocity error	Flapping frequency error	Angle error	Aircraft type	Ref
Pressure sensors	24	$0.459 m\cdot s^{-1}$ ($12\text{-}18 m\cdot s^{-1}$)	-	0.340° (AOA:-3~11°)	Fixed wing	9
	5	$0.620 m\cdot s^{-1}$ ($12\text{-}20 m\cdot s^{-1}$)	-	0.510° (AOA:-9~11°)	Fixed wing	10
	4	$0.758 m\cdot s^{-1}$ ($7\text{-}15 m\cdot s^{-1}$	-	1.792° (AOA:-4~16°)	Fixed wing	11
Flow sensors	3	$0.270 m\cdot s^{-1}$ ($5\text{-}28 m\cdot s^{-1}$)	-	0.870° (AOA:0~20°)	Fixed wing	12
	2	$0.151 m\cdot s^{-1}$ ($2\text{-}4 m\cdot s^{-1}$)	-	0.580° (AOA:-20~20°)	Fixed wing	13
Biohybrid sensor	1	$0.064 m\cdot s^{-1}$ ($1\text{-}4.35 m\cdot s^{-1}$)	0.043Hz	0.910° (Pitch:5~45°)	Flapping wing	Our

AOA: Angle of Attack

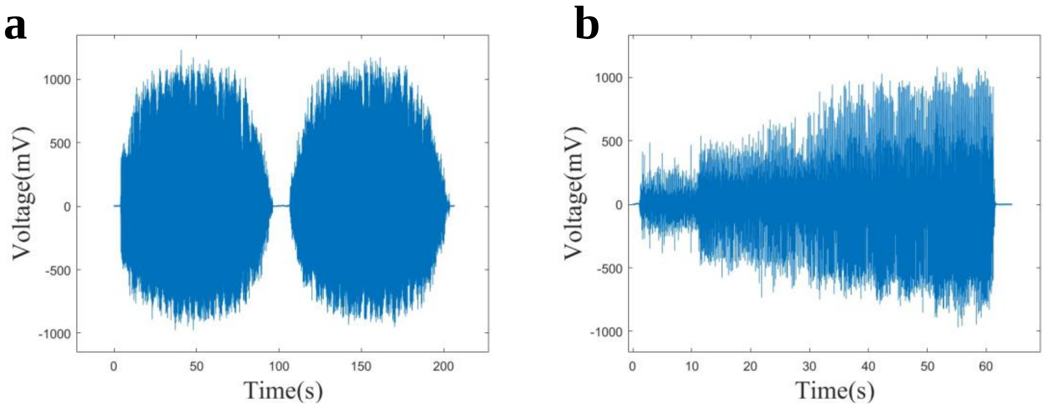

Supplementary Fig. 27: Raw signals generated by variable-frequency flapping. a Original voltage signal generated by sinewave-modulated flapping. **b** Original voltage signal generated by step-function-modulated flapping.

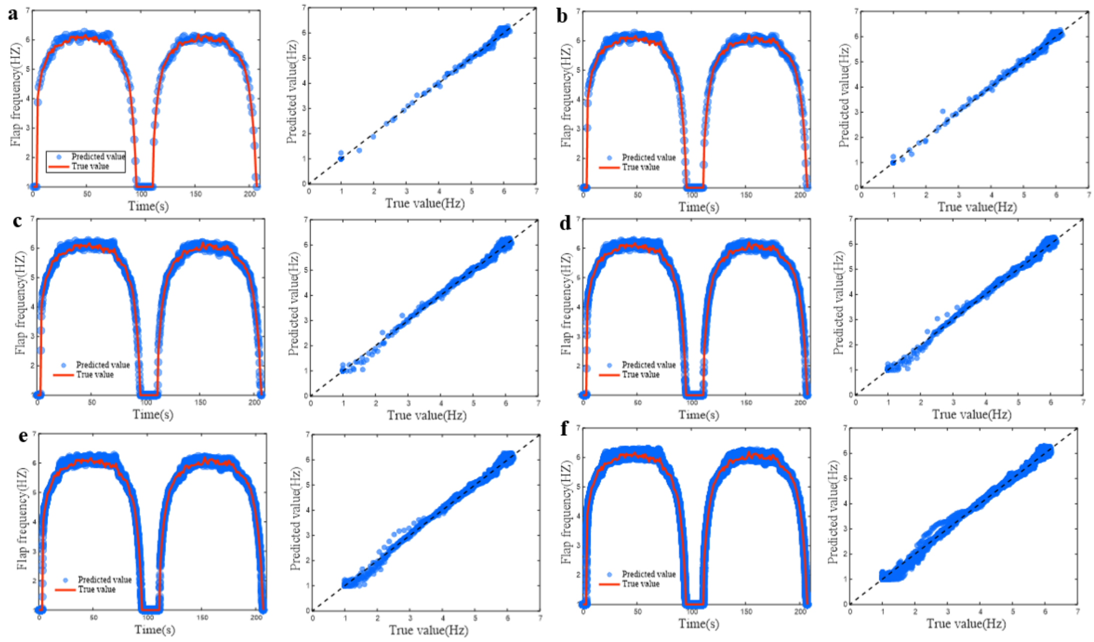

Supplementary Fig. 28: Flapping frequency recognition results at different sliding window step sizes. a Sliding window step = 1s, absolute error = 0.0746Hz. **b** Sliding window step = 0.5s, absolute error = 0.0710Hz. **c** Sliding window step = 0.2s, absolute error = 0.0713Hz. **d** Sliding window step = 0.1s, absolute error = 0.0714Hz. **e** Sliding window step = 0.05s, absolute error = 0.0717Hz. **f** Sliding window step = 0.01s, absolute error = 0.0722Hz.

10.Feathered flapping wing robot and transfer learning

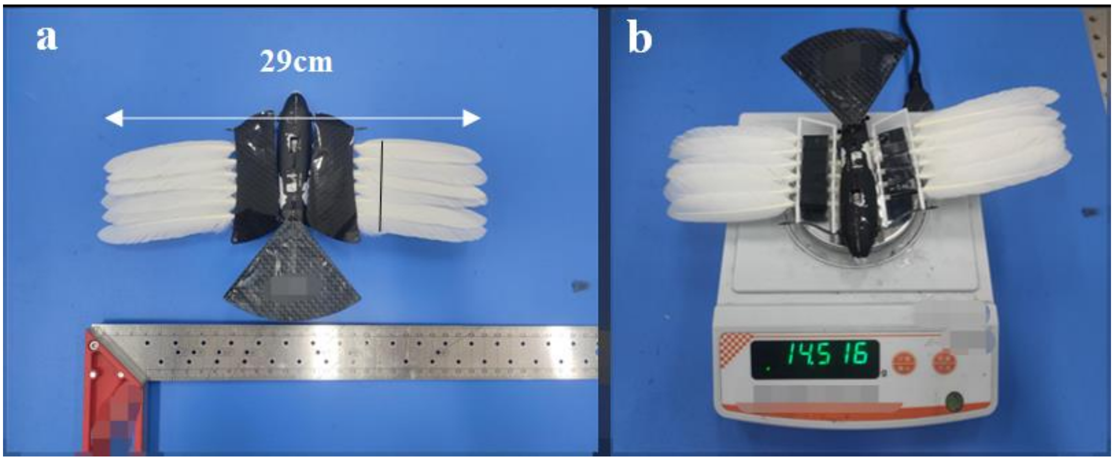

Supplementary Fig. 29: Display and parameter description of the feathered flapping wing robot. a Dimensions of the feathered flapping wing robot. **b** Weight of the feathered flapping wing robot.

This flapping-wing robot is adapted from the META-BIRD model by BIONICBIRD. We removed its original wings and installed custom-designed, 3D-printed attachments on both sides. One of these attachments is designed to accommodate the insertion of six feathers.

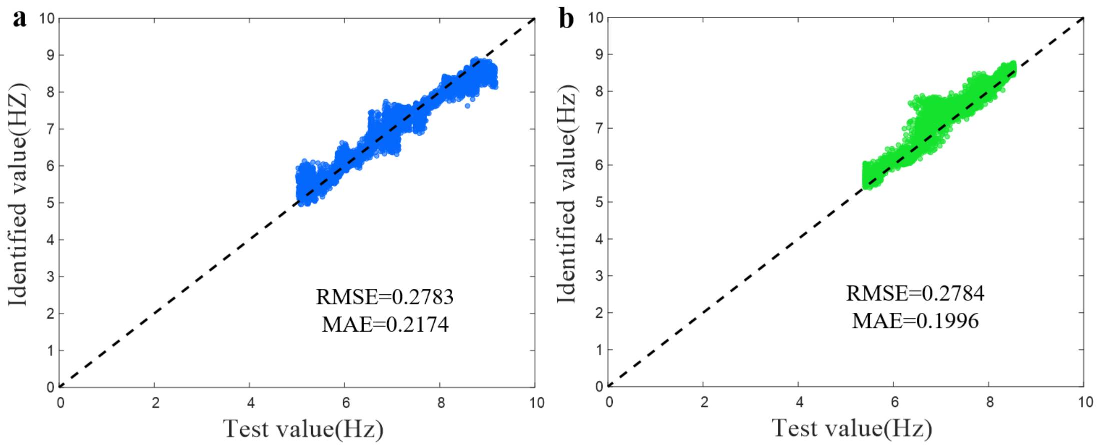

Supplementary Fig. 30: Generalization error in variable-frequency flight of the feathered flapping wing robot after transfer learning. a Generalization error for the robot's 0-100% approximately linearly varying frequency flight. **b** Generalization error for the robot's irregularly varying frequency flight.

Supplementary Fig. 30 presents the recognition errors for variable-frequency flight shown in the main Fig. 6e. The recognition was performed using the new network

obtained after transfer learning (Fig. 6d). Observing the error distribution, it is primarily concentrated around the dashed line with a slope of 1, indicating a high alignment between test values and recognition values. The root mean square error (RMSE) for the 0-100% approximately linearly varying frequency flight sequence is 0.28 with an average error of 0.22, while for the irregularly varying frequency flight sequence, the RMSE is 0.28 with an average error of 0.20.

Due to the input format of the network structure, which consists of 1000 data points (1s time length), the recognition process results in a single flapping frequency value for each second. In the mechanical bird's variable-frequency flight experiment, the initiation and termination of flapping involve high acceleration, causing the recognition values to lack information about the initial acceleration and final deceleration segments within 1s. This issue could be addressed by reducing the network input length. When the input time length is sufficiently small, the network can recognize flapping frequencies in smaller time units, capturing the high acceleration segments and improving recognition resolution. However, this problem is not the focus of the current study.

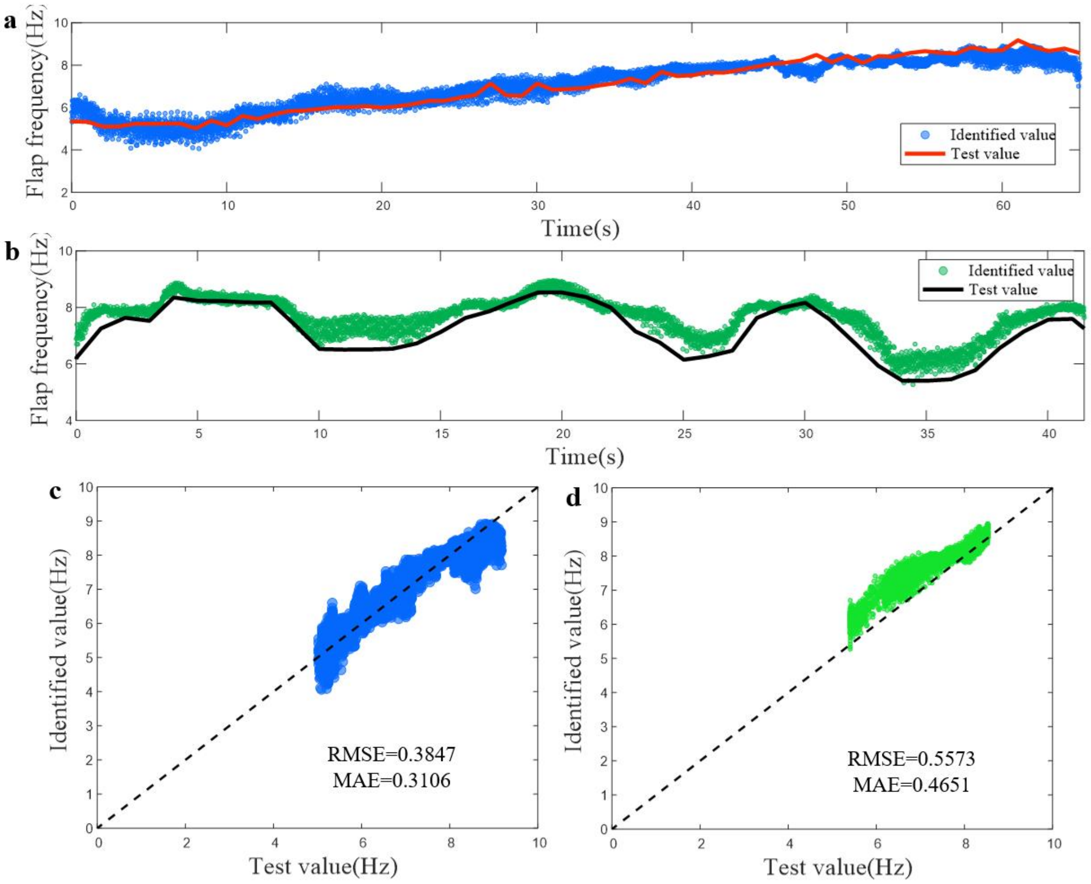

Supplementary Fig. 31: Generalization results and errors in variable-frequency flight of the feathered flapping wing robot recognized by the original network without transfer learning. a Comparison between test values and recognition values for the robot 's 0-100% approximately linearly varying frequency flight sequence, number of samples n = 6501. **b** Comparison between test values and recognition values for the robot 's irregularly varying frequency flight sequence, number of samples n = 4151. **c** Generalization error for the robot 's 0-100% approximately linearly varying frequency flight. **d** Generalization error for the robot's irregularly varying frequency flight. Source data are provided as a Source Data file.

The comparison chart in Supplementary Fig. 31 presents the test values versus the recognition values obtained through the identification using the original network (Fig. 4i), rather than the newly trained network after transfer learning fine-tuning (Fig. 6d). Computing the errors, the root mean square error for the linearly varying frequency flight test values and recognition values in the range of 0-100% is 0.3847, with an

average error of 0.3106. For the irregularly varying frequency flight sequence test values and recognition values, the root mean square error is 0.5573, with an average error of 0.4651. Contrasting with the recognition accuracy after transfer learning (Supplementary Fig. 30) indicates that transfer learning can effectively enhance the recognition accuracy.

11.Classification of wing morphology

Supplementary Table 5. The variables for feature extraction

The variables for feature extraction				
Maximum	Minimum	Mean	Median	Peak-to-peak value
Rectified mean	Variance	Standard deviation	Kurtosis	Skewness
Root mean square	Mean square	Root mean square	Waveform factor	Crest factor
Impulse factor	Clearance factor	Amplitude centroid frequency	Mean frequency	Root mean square frequency
Frequency variance	Frequency standard deviation	Spectral kurtosis mean	Spectral kurtosis standard deviation	Skewness of spectral kurtosis
Kurtosis of spectral kurtosis				

After dimensionality reduction using Principal Component Analysis (PCA), we present the cumulative explained variance contribution rates for the first eight principal components, with a total contribution rate reaching 95%. The blue squares represent the contribution rate of each principal component, and the blue dashed line reflects the cumulative contribution rates of the first eight principal components. Extracting the first three principal components, we visualize the distribution of characteristic features of three wing signals in three-dimensional space.

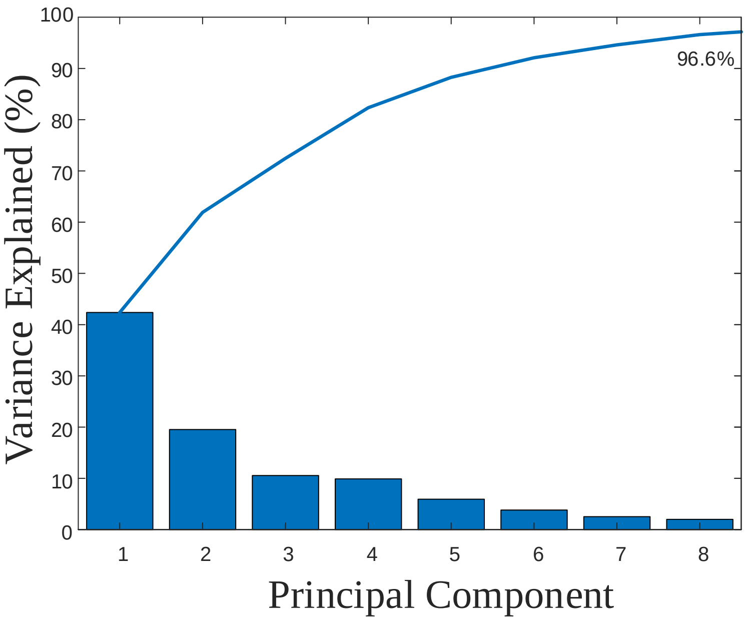

Supplementary Fig. 32: The principal component distribution, obtained through Principal Component Analysis (PCA), exhibits a cumulative explained variance contribution rate exceeding 95%.

12.Practical flight experiments

The motion capture system used in our indoor experiments has a hexadecagon framework with a side length of 0.98 m, a circumcircle diameter of 5.12 m, and a height of 3.52 m, with a green screen used for external coverage. Eight MER-160-227U3M black-and-white cameras were used for capturing, each with a resolution of 1440×1080p, equipped with Sony IMX273 CMOS sensors, and having an effective area of 1/1.29 inches. The cameras were fitted with Myutron FV0420 lenses, featuring a focal length of 4.16 mm and a maximum aperture of F/2.0. The eight cameras were evenly distributed in a crossed pattern, with four positioned at a higher level (1.95 m) and four at a lower level (0.40 m), operating at a frame rate of 100 Hz.

During actual flight, due to the frame rate limitations, it is challenging to precisely identify instantaneous flapping frequencies. Therefore, we collected the voltage signals generated during flight and performed FFT (Fast Fourier Transform) on these signals to determine the primary frequency, which was then used as the flapping frequency at that moment. The flight speed was provided by the motion capture system. Using the intrinsic and extrinsic parameters obtained through calibration of the cameras, we placed white markers on the head and tail of the flying device. The position of these markers in each frame of the flight video was recorded using the MATLAB APP DLTdv8a, allowing for the reconstruction of the three-dimensional space. By obtaining the displacement data of the head and tail markers in the **X**, **Y**, and **Z** directions and calculating the pitch angle and displacement increments per unit time, we determined the speed, with the unit time being the reciprocal of the frame rate. The time series pitch Angle and velocity data are processed by moving mean and used as the final test values. During indoor flights, the aircraft performs maneuvers such as turning and ascending, occasionally moving out of the camera array's capture range. Consequently, the tested values for flight speed and pitch angle may exhibit interruptions. We selected flight samples that were fully captured by the camera array as our training set and used data independent of the training set as the test set for perception validation. For outdoor

flight, due to the difficulty of outdoor monitoring and the instability of the environment, it is difficult to give the real value of the flight parameters, and smooth (Function in MATLAB) the final prediction results.

We have developed a customized ornithopter featuring a wing-flapping mechanism. The wings are constructed with a PET film as the primary support layer, with PVDF-feather hybrid sensors mounted on the outermost edge of each wing. Additional feathers are layered sequentially over the PET base. Lightweight clasps are positioned along the wing's central axis to ensure secure attachment and stability during flapping. Wing motion is driven by a gear assembly and a miniature motor.

A signal acquisition/wireless transmission module, measuring 2.2cm × 1.6cm × 1.6mm and weighing 1.465g, is mounted at the rear of the ornithopter and connected to the PVDF-feather hybrid sensors. Both the flight control board and the signal acquisition/wireless transmission module are powered by a single 1s lithium battery, with the entire robot weighing 28.465g. This setup enables untethered flight, as well as signal acquisition and wireless transmission. The overall operational principle is illustrated in the Supplementary Fig. 33. The first part is the flight drive module, which includes the flight control board, micro motors, and gear set to control the wing flapping frequency. The specific component connection diagram is shown in Supplementary Fig. 34. The electronic components on the flight control board include an MCU (PAN742), IMU (BMI270), Low-Dropout Regulator, and a wireless transceiver module (CC2530), all powered by a 100mAh 1S lithium battery. The second part is the biohybrid sensing module, which comprises the biohybrid sensor, signal acquisition/wireless transmission circuit board, and CNN algorithm to enable the recognition of flight parameters. This module is also powered by the same 100mAh 1S lithium battery. The specific component connection diagram is shown in Supplementary Fig. 35. The electronic components on the signal acquisition/wireless transmission board include an MCU (STM32), Low-Dropout Regulator, a wireless transceiver module (CH9140), operational amplifiers, and switched capacitor inverter. The sensor is connected to the

signal acquisition/wireless transmission board via wires. Bluetooth communication is achieved using BleComManager software, with the host computer sending commands for data acquisition and wireless transmission. The sensor data is then processed by the CNN algorithm module for recognition.

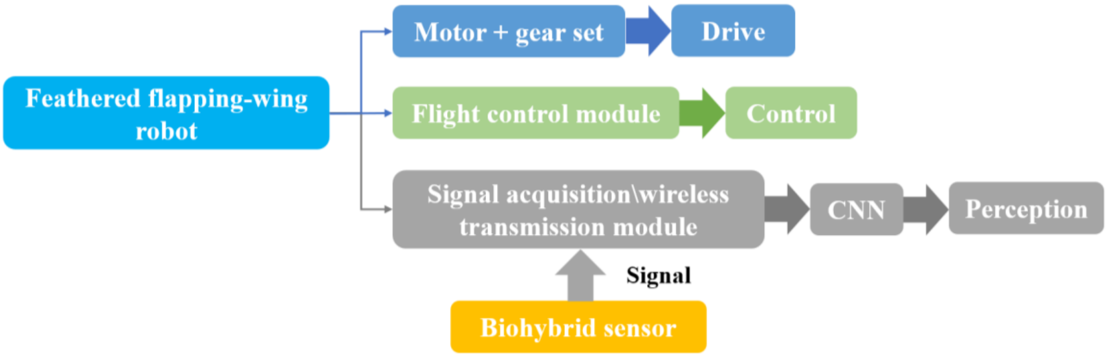

Supplementary Fig. 33: Operational principles of the flapping-wing robot.

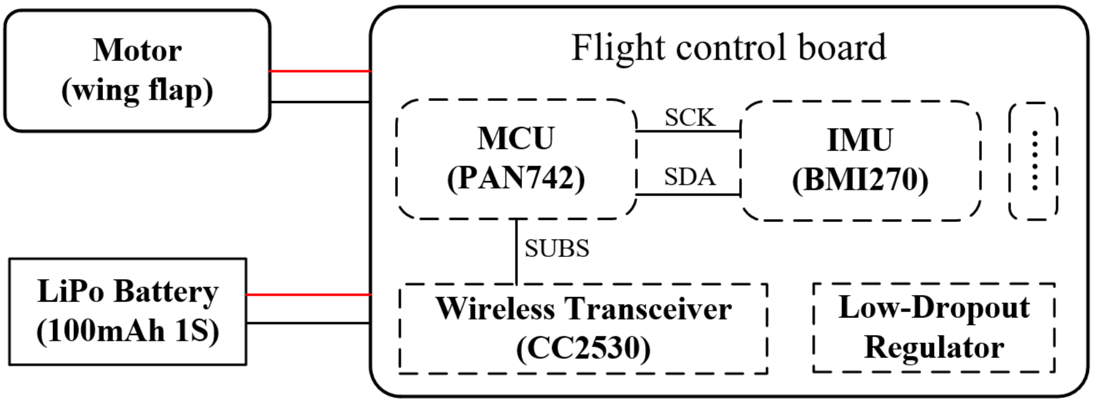

Supplementary Fig. 34: Schematic diagram of the component connections in the drive module.

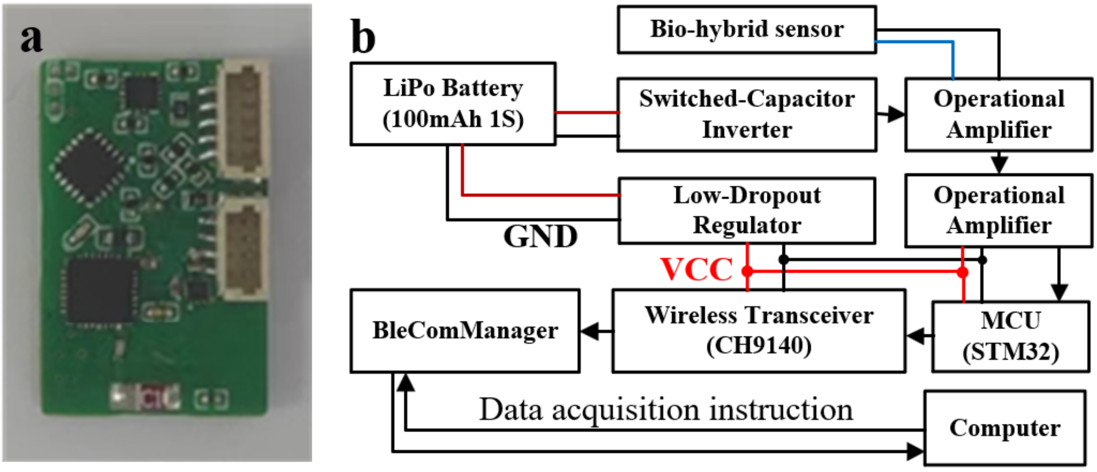

Supplementary Fig. 35: Integrated signal acquisition and wireless transmission module. a Display of modules. **b** Schematic diagram of the component connections in the perception module.

Supplementary Table 6. Mass decomposition chart for feathered flapping-wing robots

Compositional parts	Mass(g)	Percentage
PVDF	0.23	0.79%
Signal acquisition and wireless transmission module	1.47	5.15%
Conducting wire	1.83	6.44%
Feathered wings	4.45	15.63%
Fuselage (skeleton + flight control board + power supply)	20.49	71.99%
Total	28.47	100.00%

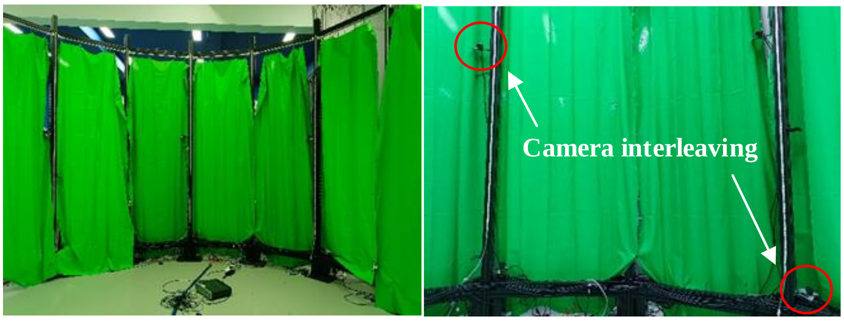

Supplementary Fig. 36: The motion capture system.

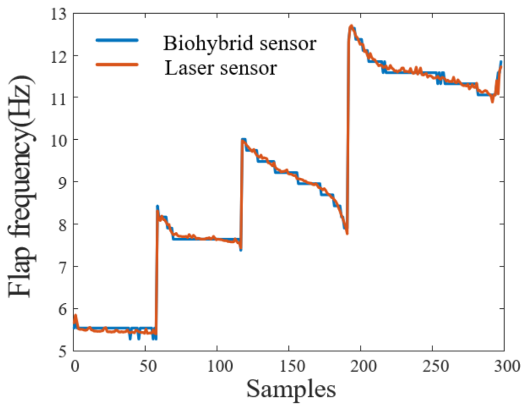

Supplementary Fig. 37: Comparison of flapping frequency monitored by the laser displacement sensor with the signal's dominant frequency, comprising 298 samples, showing the average relative error of 0.842% (number of samples n = 298). Source data are provided as a Source Data file.

Supplementary References

1. Zhang, F., Jiang, L. & Wang, S. Repairable cascaded slide-lock system endows bird feathers with tear-resistance and superdurability. *Proc. Natl. Acad. Sci. U. S. A.* **115**, 10046-10051 (2018).
2. Martins, P., Lopes, A. C. & Lanceros-Mendez, S. Electroactive phases of poly(vinylidene fluoride): Determination, processing and applications. *Prog. Polym. Sci.* **39**, 683-706 (2014).
3. Lv, F. et al. In-situ electrostatic field regulating the recrystallization behavior of P (VDF-TrFE) films with high fl-phase content and enhanced piezoelectric properties towards flexible wireless biosensing device applications. *Nano Energy*. **100**, 107507 (2022).
4. Ren, J. et al. Enhanced dielectric and ferroelectric properties of Poly(vinylidene fluoride) through annealing oriented crystallites under high pressure. *Macromolecules*. **55**, 2014-2027 (2022).
5. Tan, T., Yan, Z. & Hajj, M. Electromechanical decoupled model for cantilever-beam piezoelectric energy harvesters. *Appl. Phys. Lett.* **109**, 79-82 (2016).
6. Abdelkefi, A., Yan, Z. & Hajj, M. R. Modeling and nonlinear analysis of piezoelectric energy harvesting from transverse galloping. *Smart Mater. Struct.* **22**, 025016 (2013).
7. Wang, Q., Goosen, J. F. L. & van Keulen, F. A predictive quasi-steady model of aerodynamic loads on flapping wings. *J. Fluid Mech.* **800**, 688-719 (2016).
8. Mukherjee, S. & Ganguli, R. Non-linear dynamic analysis of a piezoelectrically actuated flapping wing. *J. Intell. Mater. Syst. Struct.* **21**, 1157-1167 (2010).
9. Li, N. et al. A compact embedded flight parameter detection system for small soaring UAVs. *Ieee-Asme Trans. Mechatron.* **29**, 52-63 (2024).
10. Samy, I., Postlethwaite, I., Gu, D. & Green, J. Neural-network-based flush air data sensing system demonstrated on a mini air vehicle. *J. Aircr.* **47**, 18-31 (2010).
11. Wood, K. T., Araujo-Estrada, S., Richardson, T. & Windsor, S. Distributed pressure sensing - based flight control for small fixed-wing unmanned aerial systems. *J. Aircr.* **56**, 1951-1960 (2019).
12. Zhu, R., Que, R. & Liu, P. Flexible micro flow sensor for micro aerial vehicles. *Front. Mech. Eng.* **12**, 539-545 (2017).
13. Gong, Z. et al. Flexible calorimetric flow sensor with unprecedented sensitivity and directional resolution for multiple flight parameter detection. *Nat. Commun.* **15**, 3091 (2024).
