# Supplementary material for: Avian-inspired embodied perception in biohybrid flapping-wing robotics
Source: Nat Commun. 2024 Oct 22;15:9099. doi: 10.1038/s41467-024-53517-6 (PMC11496644; doi:10.1038/s41467-024-53517-6)
Supplement: Supplementary file 2 — Description of Additional Supplementary Files [file 41467_2024_53517_MOESM2_ESM.pdf]

Description of Additional Supplementary Files

Supplementary Movie 1: Feather-PVDF biohybrid perceptual structure introduction

Supplementary Movie 2: Peeling experiment

Supplementary Movie 3: Fatigue experiment

Supplementary Movie 4: Bending experiment

Supplementary Movie 5: Motion and environmental perception experiments

Supplementary Movie 6: The data preprocessing, training methodology and presentation of results

Supplementary Movie 7: The real-time identification of flapping frequency in feathered flapping-wing robot

Supplementary Movie 8: The real-time identification of wind speed in feathered flapping-wing robot

Supplementary Movie 9: The real-time identification of pitch angle in feathered flapping-wing robot

Supplementary Movie 10: The real-time identification of wing shape in feathered flapping-wing robot

Supplementary Movie 11: Experiments of untethered indoor and outdoor flight
